# A brief tablet-based intervention benefits linguistic and communicative abilities in toddlers and preschoolers

**DOI:** 10.1038/s41539-024-00249-3

**Published:** 2024-05-30

**Authors:** Marcela Peña, Constanza Vásquez-Venegas, Patricia Cortés, Enrica Pittaluga, Mitzy Herrera, Esteban J. Pino, Raul G. Escobar, Ghislaine Dehaene-Lambertz, Pamela Guevara

**Affiliations:** 1 Cognitive Neuroscience Laboratory, School of Psychology, Pontificia Universidad Católica de Chile, Santiago, Chile; 2 National Center for Artificial Intelligence CENIA FB210017, Basal ANID, Santiago, Chile; 3 Faculty of Engineering, Universidad de Concepción, Concepción, Chile; 4 Neonatology Department, Complejo Asistencial Dr. Sótero del Río, Santiago, Chile; 5 Pediatric Neurology Section, Medical School, Pontificia Universidad Católica de Chile, Santiago, Chile; 6 Cognitive Neuroimaging Unit, CNRS ERL 9003, INSERM U992, CEA, Université Paris-Saclay, NeuroSpin Center, Gif/Yvette, France

**Keywords:** Education, Human behaviour

## Abstract

Young children’s linguistic and communicative abilities are foundational for their academic achievement and overall well-being. We present the positive outcomes of a brief tablet-based intervention aimed at teaching toddlers and preschoolers new word-object and letter-sound associations. We conducted two experiments, one involving toddlers ( ~ 24 months old, *n* = 101) and the other with preschoolers ( ~ 42 months old, *n* = 152). Using a pre-post equivalent group design, we measured the children’s improvements in language and communication skills resulting from the intervention. Our results showed that the intervention benefited toddlers’ verbal communication and preschoolers’ speech comprehension. Additionally, it encouraged vocalizations in preschoolers and enhanced long-term memory for the associations taught in the study for all participants. In summary, our study demonstrates that the use of a ludic tablet-based intervention for teaching new vocabulary and pre-reading skills can improve young children’s linguistic and communicative abilities, which are essential for future development.

## Introduction

Toddlers and preschoolers exhibit several important linguistic capacities that serve as predictors for their future success in both verbal and written linguistic skills. Among these predictors are size of vocabulary^1^, knowledge of the sound of letters^2–4^, and the ability to vocalize^5^. These capacities are influenced by various factors, one of which is socio-economic status (SES). A previous study revealed that children from low-income families have approximately 30 million fewer words addressed to them than their counterparts from higher SES backgrounds by the time they reach their third year of age^6,7^. Many low SES-related factors are stressful and may explain this gap. For instance, financial scarcity is associated with a significant suppression of the caregivers’ speech towards their children even in families with mid and high SES^8^. The word gap is noticeable as early as the 18-month age mark and is linked to smaller vocabulary, increased difficulty in identifying the visual referent of familiar words at 18 and 24 months^6^, and poorer academic performance later in life^9^. These findings underscore the significance of this preventable situation, which impacts a substantial population of children. Given this context, the use of new technologies could play a pivotal role in fostering vocabulary acquisition from a very young age, particularly in situations where there is a shortage of resources for linguistic stimulation.

To contribute to this subject, we conducted two experiments to assess the impact of a tablet-based intervention involving a training phase (hereafter also called as “game”) on the linguistic and communicative abilities of both toddlers (~24-month-olds) and preschoolers (~42-month-olds).

There is broad consensus about the general recommendation that before the age of 2 years, children should not be exposed to non-interactive screen devices such as television or DVDs, and that exposure should not exceed 2 h per day between the ages of 2 and 5 years^10,11^. A recent meta-analysis (involving 42 studies and 18,905 children) exploring the effect on language skills of screen exposure on this type of device for young children reports that more hours of screen exposure is associated with lower language skills, while better-quality screen exposure (i.e., educational programs) and co-viewing are associated with stronger child language skills^12^. In contrast, less is known about how interactive technologies may influence learning at such early ages^13,14^. Most studies conclude that more research is needed and remain cautious^15,16^ because screen-time greater than 1–5 h per day may be associated with cognitive difficulties^17^. To illustrate the efforts summarizing previous studies in preschoolers, we describe two meta-analyses. A recent review of 36 empirical articles in 0 to 5-year-old children (79 effect sizes and 4,206 participants) reported an overall positive effect of touchscreens on diverse learning (Cohen’s d = 0.46)^18^. Other reviews support the idea that interactive technologies can promote learning, but only when use is regulated in time and supervised by caregivers^19,20^.

Interactive technologies would possess several properties that make them convenient alternatives for supporting early education. Interactive technologies are appealing and engaging to young children who perceive them as play, and parents are generally well-acquainted with the functional capabilities and limitations of interactive technologies. Some studies report that parents and children are active users of interactive technologies, sharing the activity in many cases, while not perceiving the technologies as a threat to early child development^21,22^. For example, when the family supports the use of technologies to promote literacy in 3-4-year-old children, the children benefit from it^23^, and the parents are able to use the technologies to guide their children’s learning and resolve their doubts^24^. Lastly, interactive technologies are relatively cost-effective, making them advantageous for public policy initiatives in low-income countries.

The current version of our game represents the culmination of a prior, unpublished pilot study including children aged 24 to 36 months. In this initial study, the children received training involving familiar word-object associations (e.g., “ball”), uncommon word-object associations (e.g., “narwhal”), and pseudoword-object associations (e.g., “tefu” associated with an entirely novel object).

To train vocabulary, we adopted the fast-mapping paradigm^25–29^. Fast mapping is a word-learning process that creates an initial association between a new word and its meaning. We selected fast mapping to design our game because previous studies have shown that toddlers and preschoolers succeed in this task in the short^30^ and long term^29^. Furthermore, previous studies have shown that fast mapping ability reflects information about linguistic development in young children, as it is positively associated with concurrent and subsequent language development in late-talking toddlers^31^, typically developing^31^, and atypically developing children^32^.

In our pilot study, the word-object associations were displayed on a 21-inch all-in-one touchscreen computer, and the training involved six to eight 15-min-long sessions, three times per week. The training sessions were conducted in one of three possible settings: a laboratory environment (*n* = 62), a nursery room (*n* = 18), and a foster home room (*n* = 20). The results indicated that, on average, all children successfully learned the uncommon word-object associations but struggled with the pseudoword-object associations. Interestingly, the older children frequently vocalized the words during the training. Finally, it was found that the children accurately recalled the acquired uncommon word-object associations even 4 months after their participation in the study.

In the present study, we built upon the initial groundwork by refining its methodology. Specifically, game deployment was transitioned from computer monitors to tablets, and training was exclusively concentrated on uncommon word-object associations. Furthermore, preschoolers were actively encouraged to vocalize during the study, primarily to enhance their engagement with the game. Additionally, training in letter-sound associations was introduced to gather data on children’s interest and ability to learn these types of associations, with the intention of informing future studies. Lastly, the current study was conducted exclusively in nursery room environments.

Our primary objective was to attain a greater understanding of the potential transferability of brief fast mapping training to linguistic and communicative skills, both in the immediate and long term. The training in letter-sound associations was exploratory and focused on gathering data regarding young children’s interest and ability to learn these associations. The incorporation of vocalization was primarily intended to enhance task engagement.

In this study, both, toddlers (Experiment 1, *n* = 101) and preschoolers (Experiment 2, *n* = 152) were evaluated. The linguistic and communicative gains of the children who played the game (Study group) were compared with those of a Control group (Supplementary Fig. 1). The Control group consisted of children who were carefully matched with children in the Study group and did not participate in the game during the comparison period. Before and after the Study group played the game, we assessed the linguistic and communicative abilities in all children by applying standard assessment batteries. The intervention had thus a pre-training evaluation, a training, and a post-training evaluation periods. In Experiment 1, the assessment batteries were the Ages and Stages Questionnaires^33,34^ (hereafter ASQ-3) and the McArthur-Bates’ Communicative Development Inventory^35,36^ (hereafter CDI). In Experiment 2, we applied the ASQ-3, the Test for Auditory Comprehension of Language^37^ (hereafter TECAL), and the Test Evaluating Phonological Simplification Processes^38^ (hereafter TEPROSIF) (see Methods). After the post-training evaluation ended, we measured how much each child gained in language and communication whether she played the game or not and compared these gains. Finally, the children from the Control group could play after the intervention finished and we analyzed its game performance.

**Fig. 1:**
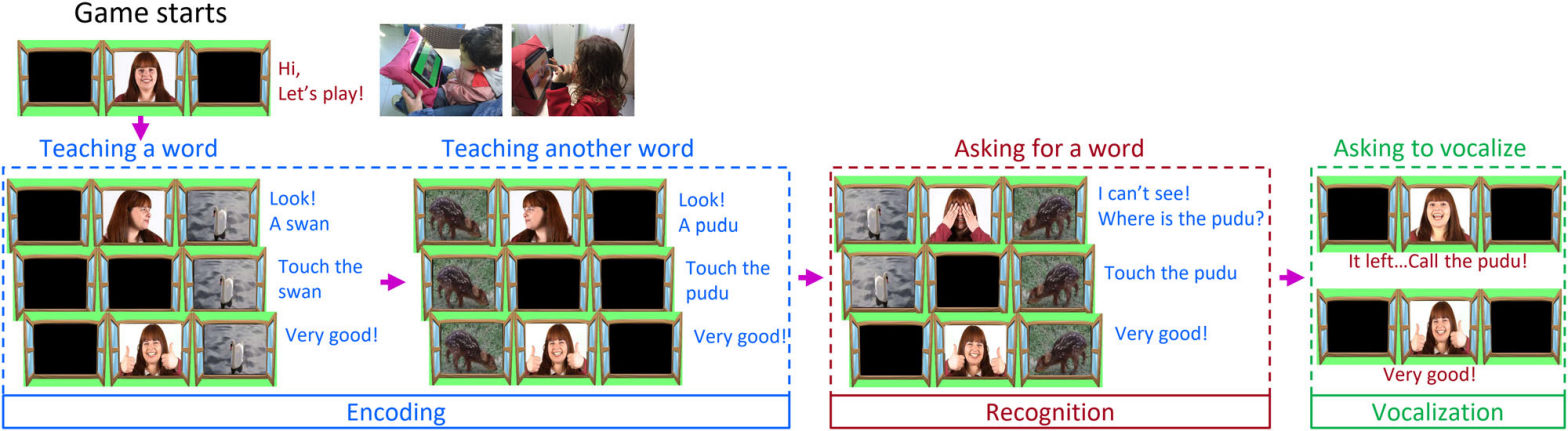
Schematic trial structure. The game was guided by videos featuring an early child educator (hereafter guiding educator). All the trials in both Experiments followed a similar structure. Here, a word-object association trial is illustrated and explained as an example. The encoding phase consisted of the consecutive training of two word-object associations with the respective accompanying visuals displayed on opposite sides of the screen. The guiding educator directed the child’s attention with interactive language, such as “Look! a [word], touch the [word].” Subsequently, the child’s tactile response was classified as omitted, correct, or incorrect, following the criteria outlined in the Methods section. During the recognition phase, both of the trained images were simultaneously displayed on the screen. The guiding educator then prompted the child to select the image corresponding to one of the two priorly taught words, using phrases like “Uh oh, where is the [word]? Touch the [word].” The child’s tactile response was then evaluated and categorized accordingly. For preschoolers (Experiment 2), the trial extended to the vocalization phase. Here, the guiding educator encouraged the child to vocalize the word being tested. This phase employed prompting such as “Uh oh, it’s gone. Call the [word].” Correct answers received positive social reinforcement, including applause and expressions of praise. Omitted and incorrect responses received encouraging messages such as “Let’s try again.” The trial structure remained consistent for the letter-sound association task, except for one variation. In this task, correct responses during the recognition phase were acknowledged with positive feedback that included a word beginning with the evaluated letter. For example, the guiding educator would say, “Bravo! BBB, Balloon.” The children received the tablet training individually in a quiet room setting, and the tablets were secured onto soft, cushion-like supports for convenience and comfort. At the top of the figure, pictures of both a toddler (Experiment 1) and a preschooler (Experiment 2) engaging in the game are included. We have obtained informed consent for the specific images of the adults appearing in this publication, and parental consent has been secured for the inclusion of photos featuring children.

The structure of each trial of the game is illustrated in Fig. 1. An early childhood educator (hereafter supervising educator) minimally oversaw the game sessions. Each trial consisted of two consecutive phases: an encoding phase and a recognition phase. During the encoding phase, children were tasked with learning two distinct associations, either between words and the objects they describe or between letters and their corresponding sounds. Subsequently, in the recognition phase, the children were asked to demonstrate their ability to recognize these associations by touching the image associated with one of the previously taught words or letters. In both the encoding and recognition phases, the children’s responses were categorized as either correct, omitted, or incorrect (see Methods). For preschool-aged participants (Experiment 2), each trial additionally included a vocalization phase. During this phase, the children were prompted to verbalize the word name of the object, or the sound of the letter shown on screen. The application recorded the children’s vocalizations, which were immediately evaluated by the supervising educator. Any vocalization from the preschooler that resembled a word or letter-sound was considered correct, as it demonstrated an attempt at vocalization. For these accurate responses, the application played back the child’s vocalization.

With this research design, we achieved two main objectives. First, we compared the extent of progress made by children in both the Study and Control groups concerning linguistic and communicative skills within the same time frame. Second, we examined whether and to what extent the children learned the word-object and letter-sound associations through their engagement with the game.

We hypothesized that both groups, Study and Control, would improve their communication and language skills between the pre-training and post-training evaluations, due to natural development during those evaluations. However, we anticipated that the extent of the improvement would be significantly greater for the Study group than for the Control group in both experiments. Additionally, we predicted that all participants would engage with the game and would perform above the level of chance in both the word-object and letter-sound learning tasks. Finally, we expected that the preschoolers would experience more linguistic and communicative gains than toddlers due to their age-related advanced cognitive development.

## Results

### Experiment 1

We first analyzed the linguistic and communicative gains. In the Control group, pre-post training data was collected from 39 participants for the ASQ-3 and 45 for the CDI, while in the Study group, the same data was collected from 53 participants for the ASQ-3 and 52 for the CDI. Thus, the degrees of freedom varied accordingly in the subsequent statistical comparisons. Sample sizes were similar to others previously reported in word learning studies^39,40^ (see Methods).

**Fig. 2:**
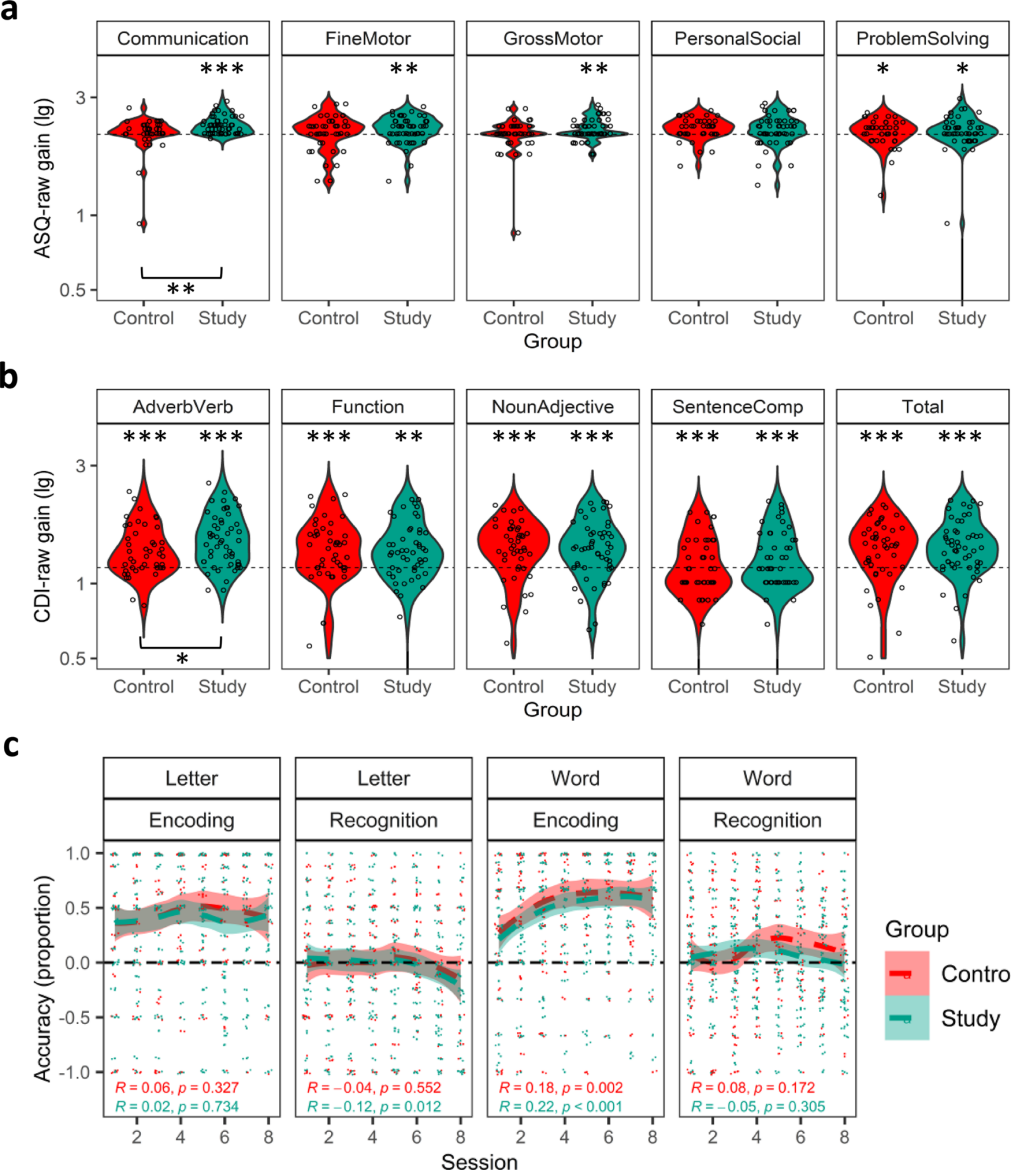
Gains in language and communication and performance in the game in toddlers. The Study group demonstrated greater standardized gains in linguistic and communicative measures than the Control group, despite both groups performing equally in the game. Plotted are for **a** each domain of the ASQ-3 and **b** each word subset of the CDI. In **a**, **b**, each circle represents the standardized gain of a participant, and the dashed horizontal lines represent null gain on the logarithmic y-axis scale. In **c**, the mean accuracy of each participant in each group is plotted for each session, task, and phase. The horizontal dashed lines represent the chance level of performance. A significant increase in accuracy with the progression of sessions is observed only in the Encoding phase for the word-object task. Although the data was analyzed from sessions one to six (the average number of sessions that the toddlers in the Control group attended), for illustrative purposes, the accuracy is plotted from sessions one to eight. Asterisks next to the boxplots indicate significant differences compared to no gain, while asterisks below the braces indicate significant differences between the groups. Significance codes: ‘*’*P* < 0.05, ‘**’*P* < 0.01, ‘***’*P* < 0.001.

The ASQ-3’s gain showed that in both groups, the standardized gains greater than zero (i.e., not null) were observed in one or more of the ASQ-3 domains (Supplementary Table 1), confirming the expected age-related progress. When comparing the groups, the Study group showed greater gain than the Control group in the raw data of the Communication domain only (Table 1 and Fig. 2a).

**Table 1:** Standardized gain in toddlers

ASQ-3	Domain	Group	n	Median gain (lg)	Interquartile rank	Z	*P*	Effect size (r)
	Communication	Control	39	2.23	0.18	−2.42	0.015*	−0.25
		Study	53	2.32	0.27			
	Gross Motor	Control	43	2.14	1.5	−1.31	0.193	−0.13
		Study	53	2.14	1.6			
	Fine Motor	Control	43	2.29	0.27	−0.59	0.552	−0.06
		Study	53	2.32	0.26			
	Problem Solving	Control	42	2.26	0.35	−0.04	0.973	−0.003
		Study	51	2.14	0.32			
	Personal Social	Control	42	2.29	0.26	0.55	0.582	0.06
		Study	51	2.29	0.27			
**CDI**	**Subset**	**Group**	**n**	**Mean gain (lg)**	**CI 95%**	***T***	***P***	**Effect size (Cohen d)**
	Adverb Verb	Control	45	1.38	0.11	−2.21	0.030*	−0.45
		Study	52	1.54	0.10			
	Function	Control	45	1.41	0.12	0.77	0.441	0.16
		Study	52	1.35	0.12			
	Noun Adjective	Control	45	1.39	0.12	−0.07	0.941	−0.01
		Study	52	1.39	0.11			
	Sentence Comprehension	Control	45	1.17	0.12	−0.75	0.457	−0.15
		Study	52	1.23	0.11			
	Total	Control	45	1.41	0.11	−0.37	0.712	−0.07
		Study	52	1.44	0.10			

The standardized gains in the ASQ-3’s five domains and in the CDI’s total scores and word subset scores (see Methods) were statistically compared. The Wilcoxon-Mann-Whitney Test was employed to compare the gains in the ASQ-3 because the data were non-normally distributed, and two independent sample *t*-tests were used for the CDI comparisons because the data were normally distributed.

Signif. Codes: 0 ‘***’ 0.001 ‘**’ 0.01 ‘*’ 0.05. Uncorrected *P*-values for planned comparisons.

Furthermore, the Study group exhibited a greater advantage when gains were compared based on category changes. In the pre-training evaluation, there were 13 (28.3%) and 20 (36.4%) toddlers classified as at-risk or having a delay in the ASQ-3 Communication Domain in the Control and Study groups, respectively. In the post-training evaluations of the Control group, within the Communication domain, four toddlers improved in category, two dropped in category, and 28 remained at the same level. In contrast, in the Study group, 15 toddlers improved, none dropped, and 38 remained at the same level (Fisher exact probability = 0.026). These results confirmed that the Study group demonstrated greater gains in the ASQ-3’s Communication Domain.

The CDI’s gain indicated that both groups showed similar baseline age-related progress across all word subsets (Supplementary Table 1). When comparing the groups, however, the Study group showed greater gains, particularly in the Adverbs and Verbs subset (Table 1 and Fig. 2b), unveiling a subtle yet significant advantage in favor of the Study group.

We submitted our gain data to multiple regression and ANCOVA analyses to measure whether others than the intervention factor explained our results. Through a comprehensive analysis involving robustbase multiple regressions (which controlled by outliers) and ANCOVA, we showed that toddler’s age, toddler’s sex, mother’s age, mother’s education and pre-training evaluations differences did not account for the greater gains in the ASQ-3 Communication scores and the CDI Adverb and Verb scores that were observed in the Study Group (Supplementary Table 2a, b). These results support the existence of a causal relationship between receiving the training intervention and achieving greater gains in communicative and linguistic abilities.

The analysis of the game data showed the following results. Although the Control and Study groups played the game at different times, a comparative analysis of their game performance over both short and long-term periods was conducted. This approach enabled the evaluation of potential disparities in the groups’ overall learning and playing abilities.

Participants in the Control and Study groups participated in 6.36 ( ± 1.87) and 7.74 ( ± 0.81) sessions, respectively. Both the letter-sound task and the word-object task showed reliability in the encoding phase, with an average split-half reliability alpha of 0.92 and 0.95, respectively.

Regardless of response accuracy, toddlers from both groups consistently responded to the tasks in the majority of trials, indicating that the game was effectively engaging (Supplementary Fig. 2).

The accuracy data, which did not follow a normal distribution, were analyzed with non-parametric longitudinal data analyses, while the response time data were analyzed with parametric repeated measure analyses, as they did follow a normal distribution. Both accuracy and response time demonstrated homogeneity of variance between the Control and Study groups.

We submitted accurate data to a series of non-parametric longitudinal data analysis. One analysis evaluated in the letter-sound and word-object tasks, separately, the effect of Phase (Encoding and Recognition) and Session (1 to 6) as within-subject factors, and Group (Study and Control) as a between-group factor. A second analysis, evaluated in the encoding and recognition phases, separately, the effects of Task (Letter-sound and Word-object) and Session (1 to 6) as within-subject factors, and Group (Study and Control) as a between-group factor. A significant effect was observed for the factor Phase. The accuracy was significantly higher for the encoding phase than the recognition phase (Fig. 2c) in both the letter-sound task (Wald-Type Statistic = 131.55, df = 1; *P* < 0.001) and the word-object task (Wald-Type Statistic = 153.64, df = 1; *P* < 0.001). Additionally, significant interactions were found between various factors. Session and Task interacted significantly, as accuracy increased across sessions only in the word-object task (Wald-Type Statistic = 31.53, df = 5; *P* < 0.001). Likewise, the interaction between Session and Phase revealed that the accuracy increased across sessions only in the encoding phase (Wald-Type Statistic = 14.29, df = 5; *P* = 0.014) (see Supplementary Tables 3 and 4).

Within response time analysis, Phase and Task demonstrated a significant interaction (F(1,68) = 4.70, *P* = 0.034, ŋ2 = 0.07). The response time was only shorter during the encoding phase as compared to the recognition phase for word-object trials only. This result indicates that for toddlers, encoding phase may be easier than recognition of word-object associations.

Importantly, no significant effects or interactions involving the factor Group were found, showing that both groups similarly handled the challenges of the game, despite playing at different times.

Additionally, we evaluated the long-term memory of the children who played the game. Four to six months after the intervention concluded, we evaluated a subset group of toddlers’ memory of the letter-sound and word-object associations that were successfully learned during the intervention (see Methods). Eleven toddlers from the Control group and 16 from the Study group were evaluated. Both groups performed significantly above chance in the word-object task (65.14% for the Control group and 62.38% for the Study group) and in the letter-sound task (69.19% for the Control group and 70.54% for the Study group). No significant differences were observed between the groups in either the word-object trials (*P* > 0.570) or letter-sound trials (*P* > 0.77). These findings confirm that the toddlers in both groups demonstrated long-term memory abilities. Notably, for the word-object associations, the learning occurred after a single exposure.

We submitted our data to a series of bivariate correlations, to explore the relationship between the game performance and the linguistic gains. No significant correlations were observed between game accuracy and standardized gain in the ASQ-3’s Communication Domain scores or the CDI’s Adverbs and Verbs scores in any comparison (*P* > 0.21 for the Control group and *P* > 0.12 for the Study group). This indicates that the intervention may have trained mainly domain-general abilities, which are relevant to language and communication.

The results of Experiment 1 indicate that certain linguistic and communicative abilities in toddlers improved through gameplay. Experiment 2 explored whether preschoolers experienced similar benefits.

### Experiment 2

Children with pre- and post-training ASQ-3 data were collected for 70 preschoolers in the Control group and 51 in the Study group. TECAL and TEPROSIF pre- and post-training data were collected for 76 preschoolers in the Control group and 68 in the Study group. The degrees of freedom for statistical comparisons varied accordingly. All gain data were distributed non-normally.

The ASQ-3’s gain revealed that both groups showed standardized gains significantly greater than zero in only the Gross Motor ASQ-3 Domain (Supplementary Table 5a), with no significant differences in any comparison between the Groups (*P* > 0.292).

**Fig. 3:**
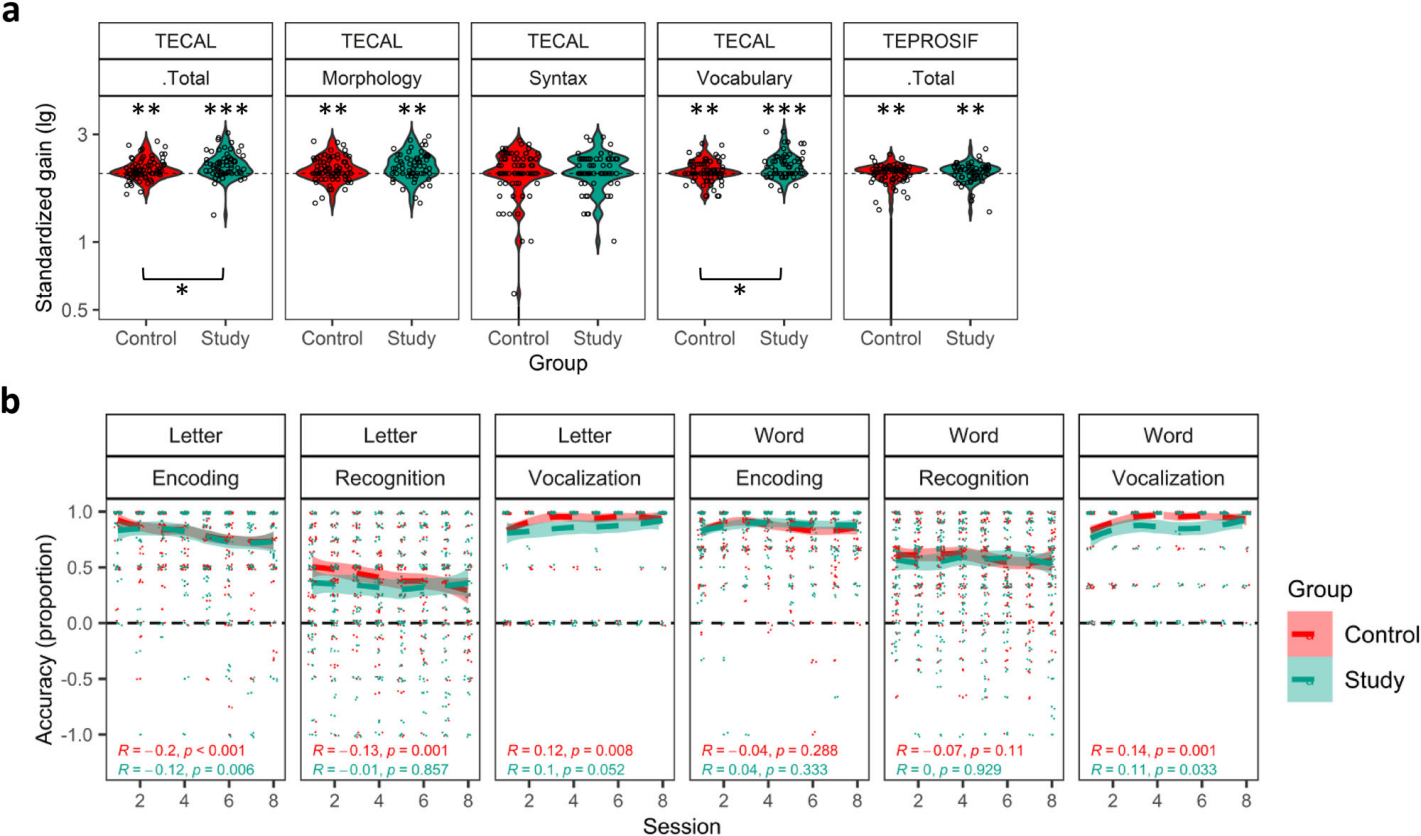
Gains in language and communication and performance in the game in preschoolers. The Study group demonstrated higher standardized gains in speech comprehension than the Control group, while both groups exhibited similar performance in the game. In **a** we plot the mean gains in the raw data of the TECAL sub-domain scores and total scores, and the TEPROSIF total scores. Each circle represents the standardized gain of a participant, and the dotted horizontal line indicates zero gain in the y-axis logarithmic scale. Except for the TECAL Syntax subset, the gain observed for each subset plotted was significantly greater than null gain, which was expected due to age-related improvements. Furthermore, the Study group showed a greater gain than the Control group in the TECAL Vocabulary subset and total scores. Illustrated in **b**, both groups displayed similar mean accuracy in the encoding and recognition phases, which remained above the chance level across sessions and tasks. Additionally, both groups similarly increased their vocalizations across sessions, indicating that speaking was an engaging and motivating activity for preschoolers. Although the data were analyzed from sessions one to seven, for illustrative purposes, accuracy is plotted from sessions one to eight. The asterisks next to the boxplots indicate significant differences compared to null gain, while the asterisks below the braces indicate significant differences between the groups. Significance codes: ‘*’: *P* < 0.05, ‘**’: *P* < 0.01, ‘***’: *P* < 0.001.

The gains in TECAL and TEPROSIF showed that both groups showed standardized gains greater than zero in both TECAL and TEPROSIF, indicating similar age-related progress during the intervention period (Supplementary Table 5b). When comparing the groups, the Study group had significantly greater standardized gain than the Control group in the TECAL vocabulary subscale and total scores (Table 2 and Fig. 3a). Differences between groups for categories (at-risk, delay, normal) were not found in TECAL (Fisher’s exact probability > 0.638) or TEPROSIF (*P* = 0.702).

**Table 2:** Standardized gains in preschoolers

Battery	Linguistic ability	Group	n	Median gain (lg)	Interquartile rank	Z	*P*	Effect size (r)
**TECAL**	Morphology	Control	76	2.04	0.31	−1.59	0.112	0.13
		Study	68	2.14	0.35			
	Syntax	Control	76	2.01	0.32	−0.15	0.884	−0.012
		Study	68	2.01	0.31			
	Vocabulary	Control	76	2.01	0.20	−2.34	0.019*	−0.12
		Study	68	2.15	0.32			
	Total	Control	76	2.07	0.23	−2.15	0.031*	−0.18
		Study	68	2.13	0.30			
**TEPROSIF**	Total	Control	76	2.06	0.17	−0.38	0.702	−0.03
		Study	66	2.06	0.21			

Standardized gain in TECAL subscales as well as TECAL and TEPROSIF total scores (see Methods). The Wilcoxon-Mann-Whitney Test was applied for comparisons because the data were non-normally distributed.

Signif. Codes: 0 ‘***’ 0.001 ‘**’ 0.01 ‘*’ 0.05. Uncorrected *P*-values for planned comparisons

The multiple regression and ANCOVA analysis showed no significant effect of bio-demographic factors and pre-training assessments in any comparison (Supplementary Table 6a, b). As in Experiment 1, these results support a causal relationship, relating the intervention’s gameplay to greater gain in speech comprehension.

The analysis of the game data showed that, on average, participants participated in 6.98 ± 1.47 and 7.26 ± 0.93 sessions, in the Control and Study group, respectively. The letter-sound and word-object association tasks demonstrated good reliability in the encoding phase (average split-half reliability alpha = 0.84 and alpha = 0.86, respectively). All preschoolers highly engaged with the game (Supplementary Fig. 3).

As in Experiment 1, we applied non-parametric longitudinal data analyses for the accuracy data because they were non-normally distributed, and parametric repeated measure analyses for response time data, which were normally distributed.

For accuracy, a main effect of Phase was observed in both the letter-sound (Wald-Type Statistic = 290.73, df = 1; *P* < 0.001) and word-object tasks (Wald-Type Statistic = 186.28, df = 1; *P* < 0.001), as the accuracy was significantly higher for the encoding phase than the recognition phase for each task (Fig. 3b). Moreover, a significant interaction was found between Session and Task; accuracy increased across sessions only for the word-object task (Wald-Type Statistic = 22.96, df = 6; *P* < 0.001), (Supplementary Tables 7 and 8).

In response time analyses, a main effect of Task was detected (F(1) = 9.75, *P* = 0.002, ŋ^2^ = 0.08), as response time was shorter for the word-object task than for the letter-sound task. Similarly, the analyses showed a main effect of Phase (F(1) = 18.55, *P* < 0.001, ŋ2 = 0.15) because the response time was shorter in the encoding phase than the recognition phase.

Again, no significant effect or interaction involving the factor Group was observed, indicating that both groups performed similarly in the game.

As in Experiment 1, 31 and 27 preschoolers in the Control and Study group, respectively, were evaluated for long-term memory of the associations learned during the intervention. All preschoolers performed with an accuracy significantly above the chance level (50%) in both the word-object task (78% and 77% for Control and Study group, respectively) and the letter-sound task (63.97% and 64.14% for Control and Study group, respectively). No significant differences between the groups (at *P* > 0.070) were found, indicating that the groups did not differ in their long-term memory capacities.

The game results indicate that the preschoolers of the Control and the Study groups showed similar capacities to engage with the game and learn from it in both, at short and long term.

The bivariate correlation analysis revealed a significant correlation in the Study group showing that higher standardized gain in TECAL total score correlated with higher mean accuracy in the encoding phase of the letter-sound task (Pearson correlation coefficient = 0.34, *P* = 0.005) (Supplementary Fig. 4). These results support the idea that the game benefits general-domain mechanisms, which are important for linguistic tasks.

Together, the results indicated that the game benefitted certain linguistic abilities of the preschoolers, replicating the results observed in the sample of toddlers in Experiment 1.

### Cross-experiments analysis

Given that both experiments pursued similar goals, applied almost identical protocols, measured equivalent language and communication variables, and evaluated children with similar bio-demographic backgrounds, their gains and game performances were compared for analysis. This analytic approach allowed for more mature conclusions about whether a tablet-based intervention benefits language and communication in young children.

The analysis of the gain across experiments was computed as follow. Since the gains were standardized in both experiments, we computed a “total gain” variable by averaging the gains in the variables that showed statistical differences between groups, in each experiment. This corresponded to the average of the gain in the ASQ-3’s Communication Domain and CDI’s Adverbs and Verbs subset in Experiment 1, and the TECAL Vocabulary and TECAL total in Experiment 2. The total gain values were submitted to a non-parametric Wilcoxon Mann-Whitney test with Experiment (Experiment 1 and Experiment 2) and Group (Control and Study) as between-subject factors.

We found a main effect of Group (*Z* = −3.56, *P* < 0.001, *r* = −0.22), as the Control groups showed less total gain than the Study groups. A main effect of Experiment was also found, as toddlers showed less total gain than preschoolers (*Z* = −8.00, *P* < 0.001, *r* = −0.51). This demonstrates that preschoolers benefitted more from the game, likely because they had more mature skills to contingently respond to interactive technologies.

We analyzed the game performance across experiments as follow. The accuracy data were submitted to a series of non-parametric longitudinal data analyses with Session (1 to 6) as within-subject factor and Experiment (Experiment 1 & Experiment 2) and Group (Control & Study) as between-subject factors. We observed a main effect of Experiment (at *P* < 0.001 in each comparison) because the accuracy was higher in Experiment 2 in both tasks (letter-sound and word-object), during both phases (Encoding and Recognition) (Supplementary Table 9 and Table 10). Moreover, there was a significant effect of Session because the accuracy increased across session only for the encoding phase of the word-object task (*P* < 0.001) (Supplementary Table 10a). A similar but quite smaller effect was observed in the recognition phase but in the contrary sense, that is, the accuracy decreased across session in this phase, probably because the task was hard for all children (Supplementary Table 10b). Similarly, in global, the response time was also shorter in Experiment 2 than in Experiment 1 (*P* < 0.001). Together, these results showed greater accuracy and shorter response time for preschoolers as compared to toddlers, which was expected because of age-related differences. Importantly, no significant effect or interaction involving the factor Group was found in the game performance, indicating that the Control and Study groups similarly handled the challenges of the game and learned from it, although they played at different times.

Comparisons of the two experiments reinforced the evidence that playing the game was causally associated with greater gains in communicative and linguistic abilities in toddlers and preschoolers, despite their differences in neurocognitive development.

## Discussion

We believe our results are particularly relevant when discussing whether and in what ways early stimulation with interactive technologies may benefit linguistic and communicative abilities in young children. Our results may be specifically relevant for early education because several prior initiatives aimed at improving vocabulary in kindergarten have been ineffective in reducing the gap observed in children who started with poor vocabulary, likely because the methods disproportionately benefitted the already advanced children^41^.

Our interest in evaluating new ways to promote vocabulary from an early age is related to a hypothesized limited number of words that children can learn per day. If a typical 3-year-old child learns up to 10 new words per day^42^, it may be hard to double this number to compensate for the SES-related gap at older ages. Preschoolers would be more capable than older children to use their knowledge of the semantic and morphosyntactic properties of the words they already know to learn new ones^42^.

Moreover, we wanted to evaluate an intervention implemented on an interactive technology because a tablet-based game is a low-cost tool for supporting early childhood educators’ activities, it is attractive for young children, and parents and caregivers are fairly adept at using the technology. If interactive technologies are used in a regulated way, they may provide such stimulation support, serving as a useful tool for early childhood educators working in educational environments where human resources are scarce.

Our study also come to contribute with the general demand for more research in the field of causal studies of tablet-based interventions on language and communication in toddlers and preschoolers. Despite their young ages, 24-month-olds and 42-month-olds engaged in the game and succeeded in learning from it in both, the short and long term. Crucially, toddlers and preschoolers who played the game showed transfer to language and communication abilities (evaluated with standard batteries), a generalization that extends beyond training ability as the holy grail of any intervention study.

Although encouraging, our results should be interpreted with caution because further studies with larger sample sizes are mandatory to support them. The strength of our results relies on the pre-post equivalent group design, with an exhaustive process to match the groups by bio-demographic factors and linguistic scores before the training phase started, followed by a random assignment of participants to the Control and Study groups. Moreover, because both groups played the game at different times of the study, we were able to replicate the game’s results in both groups, rejecting the possibility that the greater gains found in the Study group emerged from an imbalance in the children’s interest or ability to play the game.

The most remarkable results of the study showed that a brief intervention improved linguistic and communicative abilities by advancing children toward higher categories of development (defined by assessment batteries) and higher raw scores in communicative skills in toddlers (ASQ-3 Communication and CDI Adverbs and Verbs), and higher speech comprehension scores in preschoolers (TECAL). The greater improvement in the comprehensive vocabulary of the preschoolers in the Study group as compared to the Control group may indicate greater advances in the children’s comprehension of the scope of linguistic exchanges.

Interestingly, at both ages, the language and communication gains of the Study groups were weakly correlated with performance in the game. Indeed, the accuracy in letter-sound trials positively correlated with the gain in TECAL only for preschoolers in the Study group. Previous training studies in the number cognition domain, with more extensive literature detailing computer-based interventions during childhood, indicate that the lack of direct correlations between the training and cognitive gains suggests that the transfer from training to cognition relies more on general-domain than specific-domain cognitive abilities^43^. Further studies are mandatory to evaluate this possibility for the game used in our study. Nevertheless, despite the missing direct link between training performance and gains, we dare to propose three mechanisms behind these greater gains in the Study group.

First, the systematic presentation of word-object associations may have facilitated the children’s discovery of the usefulness that speech provides for understanding an environment. Previous studies show that, from infancy, naming facilitates object individuation^44^, categorization^45^, attention toward learning associations^46^, and the discovery of the symbolic value of labels associated with abstract concepts^47^.

Second, the social cues provided by the game may have facilitated learning^48^. The guiding educator provided direct gaze, child-directed speech, and socially contingent responses, all of which are cues that promote learning during early development. Only in socially contingent interactions do toddlers successfully learn novel noun-like words^49^ and verbs^50^ and generalize them to new instances^51^. Finally, the intervention setting may have familiarized the children with the effectiveness of social cues for learning language in daily life.

The third possible mechanism is immediate memory retrieval training, which involves recognizing information immediately after exposure. In our methodology, this involved presenting the recognition phase immediately after the encoding phase. Immediate memory retrieval practice benefits word-learning in preschoolers when evaluated in single sessions^52^. A recent adult brain study reported that retrieval practice fosters learning by recurrently activating an anterior-posterior network in the hippocampus, which is involved in the encoding of specific stimuli and the generalization processes necessary to apply the stored information to new instances^53^. Interestingly, despite their high engagement and success in the encoding phase, toddlers failed in the recognition phase. Although this result requires further exploration, we may hypothesize that the retrieval process inherent to a two-alternative forced-choice task could be particularly hard for 24-month-olds or may require additional processing, such as learning consolidation during sleep, which is crucial for word learning in infants and toddlers^54^.

Another important result of our intervention was the long-term memory of the trained material, although this result must be confirmed with larger samples and higher control of target-word exposure between the training and the long-term memory test. Despite the single exposure to word-object associations during the intervention, the young children recognized them four to six months later with accuracy above 62%. Especially surprising was the 65% accuracy in 24-month-olds because they performed at chance level in the immediate recognition phase. It seems improbable that caregivers taught the low-frequency words after the intervention, given their impoverished environment, therefore sleep consolidation of the learning may have mediated the posterior success in the long-term memory tests at this age. Some studies with older children suggest that long-term effects of interventions promoting vocabulary are modest and transient^55,56^ prompting the question of whether starting earlier might achieve greater success. Indeed, known words serve as an anchor for discovering new ones from continuous speech^57^, and increasing the number of neighboring words improves their phonological representations in the lexicon^58^ and facilitates efficiency in finding the correct visual referent after hearing a word^7^. These effects create a snowball effect, rapidly enhancing young children’s speech comprehension and production. Moreover, beyond the mentioned training-related possibilities, the efficiency of the intervention may have profited from the young children’s biological windows of opportunity, which are periods of development when learning is facilitated. Indeed, language acquisition for other linguistic domains such as phonology or syntax^59,60^ is facilitated during early years.

Finally, all groups showed greater accuracy for the word-object task than for the letter-sound task. Some explanations may be that young children are less familiar with letters than with words, letter shapes are less interesting than pictures, or the phonetic domain of letters is less explicit than natural categories such as objects^61^, although 2-month-olds succeed at associating two different letter-sounds (/b/ and /g/) with different specific geometric shapes^62^. Further studies are necessary to evaluate these possibilities.

Our study had several limitations. Here we describe what we think are the four main ones. First, our size effects are medium. We believe that this could be related to the short duration of the training (only a half academic period), to the fact that the game trained only word-object and letter-sound associations (not all language features with semantic and grammatical contents), and to the small size of the samples. All these effects must be considered in further studies to evaluate the strength of the benefits we found. Second, we evaluated children from low-income families only, thus evaluating children from other SES (very low, mid, or high) would be mandatory to demonstrate the transfer of the benefits promoted by the game other contexts. Third, we did not explore deeply the children’s home environments, which must be mandatory in next studies. Fourth, because budget and pandemics restrictions we did not follow up on the children’s academic achievement, which would be suitable for further studies.

In sum, we show that it is possible to engage toddlers and preschoolers in a tablet-based game with positive consequences on communicative and linguistic capacities in both the short and long term. Although the benefits of the intervention were small, they may possess an important educative value for children who struggle with the scarcity of direct human stimulation. Hopefully, our results may also encourage research of regulated integration of interactive technologies aimed at promoting learning during early development.

## Methods

### Participants

All participants were recruited at four public preschool educational centers they regularly attended.

In Experiment 1, 125 toddlers were evaluated in the study. First, the toddlers were ranked based on their age, sex, and scores in the linguistic and communicative assessments from the pre-training evaluation. In other words, we first evaluated the children, then we ranked them upon the basis of their scores in the linguistic and cognitive assessments and age, then, we selected two girls or two boys who were neighbors in the rankings, and finally randomly assigned each of them either to the Control or to the Study group. The Control group had 62 toddlers and the Study group 63. This procedure avoided differences between the groups based on the pairing factors. The Study groups received the intervention immediately after the pre-training evaluation while the Control groups received it after the complete study ended. 24 toddlers were excluded from the analysis (16 and 8 in the Control group and the Study group, respectively), either because they did not have at least one pre- and one post-evaluation or because they participated in fewer than four training sessions. 101 toddlers who had at least one pre- and one post-assessment and played four or more sessions remained: 46 in the Control group (Mean age = 23.00 months, SD = 2.59 months, 19 female) and 55 in the Study group (Mean age = 22.80 months, SD = 2.99 months, 24 female).

All toddlers belonged to a low-middle socioeconomic class, lived in a monolingual Spanish-speaking environment, were born full-term, and had a typical development at the testing time. None had a history of family-specific language impairment or family dyslexia. Toddlers with uncorrected, diagnosed visual and auditory deficiencies or severe motor problems that interfered with the use of a touch-screen system were purposefully excluded from the study. Additionally, excluded were toddlers with severe neurodevelopmental diseases and chronic conditions that caused or predisposed them to developmental difficulties, including neonatal asphyxia, epilepsy, chromosomal disorders, and inborn errors of metabolism.

The sample size was similar to others reported in word learning studies using touch-screen techniques in toddlers e.g., Walter-Laager et al., 2017, total *n* = 66, 15 to 17 toddlers per group, four groups, age range = 23–31 months^39^; Kirkorian, Choi & Pempek, 2016, total *n* = 116, 38 to 40 toddlers per group, three groups, age range = 24–36 months^40^.

Parents provided bio-demographic data (mother’s age, mother’s education, socioeconomic level, languages spoken at home, and children’s pediatrics data), and filled out the ASQ-3 and the Word and Sentences form of CDI questionnaires.

Before comparing the final data from the groups, we confirmed that, after excluding data from children that did not meet the criteria for sessions played or assessments recorded, the groups did not differ in their bio-demographic data or their linguistic and communicative abilities from the pre-training evaluations (Supplementary Table 11). The Study and Control groups were similar in mother’s education (M = 12 years of education, range = 4 to 16 years in both groups), mother’s age (Mean = 32.02, SD = 5.48 years and M = 30.69, SD = 6.20 years, in Control and Study group, respectively) and socioeconomic level (76% of the Control group and 77% of the Study group belonged to low socioeconomic level and no family belonged to high socioeconomic level). No significant differences between the groups were found in toddlers’ bio-demographic data (*P* > 0.106), in the ASQ-3 raw data (*P* > 0.200), or in the CDI scores (*P* > 0.424) from the pre-training evaluations.

In Experiment 2, 169 preschoolers were recruited for the study: 83 in the Control group and 86 in the Study group. Three preschoolers of the Control group and 14 of the Study group were excluded from the analysis, either because they played less than four sessions or because they were missing all the pre-post training assessments, leaving 152 preschoolers; 80 from the Control group (Mean age = 39.23 months, SD = 3.08 months, 44 females) and 72 from the Study group (Mean age = 39.21 months, SD = 2.87 months, 39 females).

As in Experiment 1, before submitting the data to statistical comparisons between the groups, we proved that, after the exclusion of preschoolers and data that did not meet the requirements, the groups did not differ in their bio-demographic composition or in their linguistic and communicative abilities at the pre-training evaluations (Supplementary Table 12). The groups had similar mother’s education (Mean age = 12 years, range = 4 to 16 years), mother’s age (Mean age = 32.75 years, SD = 6.37 years and Mean age = 31.03 years, SD = 5.93 years, in Control and Study groups, respectively) and socioeconomic level (75% and 74% of the families reported low-socioeconomic level in the Control and Study groups, respectively, with the remainder classified as middle socioeconomic level). No significant difference was found between the groups in the bio-demographic data (*P* > 0.237), the pre-training scores from ASQ-3 raw data (*P* > 0.120), the TEPROSIF scores (*P* > 0.870) or the TECAL scores (*P* > 0.212).

Our research respected the principles of the Declaration of Helsinki and received approval from the Pontificia Universidad Católica de Chile, Social Sciences Faculty Ethics Committee (protocol no. 190606011, 2018/09). All the children’s parents signed a written informed consent before their children participated in the study. At the end of the intervention, the participants were thanked for their time and effort with a small gift.

### Experimental design and protocol

The study employed a pre-post equivalent group design, with a Study group and a Control group in both Experiment 1 and 2 (Supplementary Fig. 1). The intervention consisted of a pre-training, a training, and a post-training step. In the pre-training step, the baseline data of each child’s linguistic and cognitive abilities as well as their bio-demographic data was collected; in the training step, the children played the game; and in the post-training step, each child’s linguistic and cognitive abilities were re-assessed and the gain in each assessment for each child was computed. The entire study was conducted over approximately four months.

### Cognitive, communicative, and linguistic measures and gains

On average, there were 54.46 days between the pre-training and post-training evaluations in Experiment 1 and 53.32 days in Experiment 2. In both experiments, parents provided bio-demographic data (mother’s age, mother’s education, socioeconomic level, languages spoken at home, and children’s pediatrics data), and filled out different questionnaires. Moreover, in Experiment 2, speech therapists handled the pre-post evaluations, and they remained blind about the participant group assignment and specific objectives of the study. Below are the properties of the standardized assessments used in each experiment.

In Experiment 1 the assessments consisted of the Spanish versions of the ASQ-3 and the Words and Sentences form of the CDI, two standard parent-report questionnaires recognized as sensitive for estimating the emerging cognitive and linguistic abilities of young children. ASQ-3 evaluates five domains of child development from 2 to 60 months of age: Communication, Gross Motor, Fine Motor, Problem Solving, and Personal-Social Development. The CDI words and sentence is addressed to 18 to 30-month-olds toddlers and quantifies the number of words understood and spoken by the toddlers and the complexity of their vocalizations. In this study, the analysis was calculated using the total number of CDI words, the number of CDI words within each of four subsets: Nouns and Adjectives, Adverbs and Verbs, Function Words, and the Sentence Complexity.

In Experiment 2, the evaluations consisted of the ASQ-3; the TECAL, which quantifies speech comprehension abilities regarding vocabulary, morphology, and syntax; and the TEPROSIF, which estimates language expression by measuring the quality of phoneme production.

We computed the gains as follow. Before any statistical comparisons involving the gain were calculated, the internal consistency of each cognitive assessment was evaluated by computing the reliability of the raw scores at the pre-training evaluation. We considered reliable Cronbach’s alpha > 0.71 for ASQ-3 and CDI, and Cronbach’s alpha > 0.73 for TECAL and TEPROSIF.

To measure the impact of the game, the standardized gain in the raw scores of ASQ-3, CDI, TECAL, and/or TEPROSIF was computed. The standardized gain in each task was computed as follows. The pre-training score was subtracted from the post-training score, then that difference was divided by the standard deviation in the pre-training evaluation computed across all participants, regardless of group.

Additionally, the ASQ-3, TECAL, and TEPROSIF tests categorize scores based on normal developmental benchmarks for each age. The changes in category for each of these tests were analyzed. For each test, the number of children who, between the pre- and post-training evaluations, improved in category (changed from delay to normal, delay to at-risk, or at-risk to normal), dropped in category (changed from normal to at-risk, normal to delay, or at-risk to delay), and remained in the same category was quantified. Then, these numbers were compared between the Study and Control groups by applying a Chi-squared analysis (Fisher’s exact test).

### The game

The game was designed by a multidisciplinary team composed of early childhood educators, speech therapists, game designers, and cognitive neuroscience researchers. The game was adjusted and experimentally validated with other small samples of children over 2 years before this study started. The game had two applications: one for the child (the main application that controlled the game) and a second one for the supervising educator (the “tutor” application) to allow for remote monitoring and control of the training when necessary. The main application taught 14 letter-sound associations and 59 infrequently used word-object associations across four to eight training sessions (Supplementary Table 13 and Supplementary Fig. 5). Each word-object association was trained once throughout the sessions to evaluate fast-mapping after a single exposure. The parents confirmed that their children were unfamiliar with the words trained. A crucial property of the applications was their contingent interaction, delivering immediate feedback to the children’s responses.

The word-object and letter-sound trials had identical structures (Fig. 1). Each trial started with an encoding phase followed by a recognition phase and, in Experiment 2, finalized with a vocalization phase.

In the encoding phase, the in-application guiding educator taught either two word-object or two letter-sound associations during each trial. The guiding educator taught one association at a time and prompted the child to touch the image, which was displayed statically, that corresponded to the spoken word or letter. If the child touched the image between 0.5 seconds before and 2 seconds after the end of the educator’s prompt, the answer was classified as correct and scored as 1. Correct responses received positive feedback consisting of social rewards such as claps and words of encouragement, and the static image was displayed as a video. The time window to collect the tactile responses started before the end of the educator’s prompt, as the child may have recognized the image after perceiving the initial phonemes of the word. If the child did not answer within the specified time window, again the guiding educator asked the child to touch the image. If the child correctly touched the image the second time, the response was classified as correct, but received a reduced score of 0.5. If the child did not provide a tactile response after the second prompt, the trial was classified as omitted and scored as 0. If the child touched the screen outside of the image, the trial was classified as incorrect and scored as -1 or, if the incorrect response occurred after the second request to touch the screen, -0.5. The omitted and incorrect responses received encouraging messages, such as “Let’s try again.” The same procedure was used to train the second word or letter-sound, which was shown on the opposite side of the screen.

The mean accuracy was then calculated for each child in each session of the encoding phase as follows. First, the scores obtained in the first and second word-object or letter-sound associations in each trial were averaged, then the average was computed across the trials of each session. The mean response time was similarly computed for the correct and incorrect responses in each trial and session.

The recognition phase immediately followed the encoding phase. The two trained images were displayed simultaneously on the screen while the guiding educator prompted the child to touch the image corresponding to one of the two previously trained words or letter-sounds. Only one word or letter-sound was tested in each trial. The child was given a window between 0.5 s before and 2 s after the guiding educator’s prompt ended to respond. The child was given a second chance to answer if they did not answer within the first response window. If the child touched the correct image, it was then displayed as a video, and the other image disappeared. To control for biases towards a specific side of the screen, the images of each association randomly appeared on either side of the screen during the encoding and recognition phases. A similar scoring procedure as the one used for the encoding phase was applied to the recognition phase.

In Experiment 2, the trial included a vocalization phase following the recognition phase. The guiding educator prompted the preschooler to vocalize the tested word or letter-sound aloud. If the preschooler did not vocalize within 2 s, the guiding educator would repeat the prompt and give the child 7 s to respond. The application recorded any vocalization made by the preschooler. The supervising educator pressed a key on the tutor tablet to classify any word-like vocalization made by the preschooler as correct, and the application played the preschooler’s vocalization back as a reward for the correct responses. All the trials without vocalizations after two prompts were classified as omitted. Although we planned to classify any non-verbal sounds, such as screams, as incorrect, those types of responses were not observed. The accuracy in the vocalization phase was computed by dividing the number of correct vocalizations by the sum of the correct and omitted ones.

We analyzed the child’s performance in the game as follow, the mean accuracy and response time were computed for each session’s encoding, recognition, and vocalization phases of both the word-object and letter-sound tasks. The mean accuracy ranged between -1 and 1, with zero indicating the at-chance level.

Interest to play, which corresponded to the proportion of non-omitted trials, regardless of the accuracy, was analyzed to explore if the participants from the Study and Control groups differed in their engagement with the game.

The interest to play, the mean accuracy and the mean response time for the correct and incorrect responses were then submitted to different analytic tools taking into account the data distribution.

To evaluate the long-term memory, we exposed each child from both groups (at this moment both had played the game) to a version of the game that had not encoding phase and directly showed the recognition phase. The evaluated word-object and letter-sound associations were the ones that each child did successfully learn during the intervention (i.e., associations with accuracy 1 or 0.5 in either the encoding or recognition phase). The computation of the correct responses and feedback used the same procedure that the ones described during the intervention.

### Statistical approaches

Since many gain values in the cognitive, communicative, and linguistic assessments and the scores in the game performance had non-normal distribution, these values were all transformed into their logarithmic format to reduce skewness of the scores. Despite log transformation, several variables, including the scores in ASQ, CDI, TECAL and TEPROSIF observed during the pre-training evaluation, the standardized gain in ASQ and TECAL, and game’s accuracy, remained non-normally distributed. CDI’s gain and game’s response time distributed normally. We applied thus either parametric or non-parametric statistics for the comparison of these variables, because all log transformed data showed homogeneity of variance between the Control and Study groups. Thus, the log-transformed data were submitted to: (a) either a one-sample t-test (2-tails, alpha = 0.05) or Wilcoxon test to compare the cognitive and linguistic gains against zero (null gain); (b) either a two-sample Welch t-test (2-tails, alpha = 0.05) or Wilcoxon-Mann-Whitney test, to compare the cognitive and linguistic gains between the Study and Control groups; (c) either a repeated measure ANOVA or non-parametric longitudinal data analysis, to compare the performance in the game between the groups. Moreover, we applied robustbase multiple regression analysis to explore the possibility that the differences in the cognitive and linguistic gains between the groups were explained by factors such as child’s age, child’s sex, mother’s age and/or mother’s education, rather than the intervention. The “robustbase” tool was applied, because it is less sensitive to outliers. Finally, non-parametric ANCOVA allowed us to evaluate if the differences we found between the groups were due to differences in the groups already present before the training started.

Statistical tests were evaluated at *P* < 0.05 level of significance. Effect sizes were estimated using *r or* partial eta squared (*η2P*) for Wilcoxon-Mann-Whitney and ANOVA, respectively. The software R was used for the analysis.

### Reporting summary

Further information on research design is available in the Nature Research Reporting Summary linked to this article.

### Supplementary information

Supplementary Information

Reporting summary

## Data Availability

The datasets used and/or analyzed during the current study are available from the corresponding author upon reasonable request.
