# Supplementary material for: A brief tablet-based intervention benefits linguistic and communicative abilities in toddlers and preschoolers
Source: NPJ Sci Learn. 2024 May 30;9:38. doi: 10.1038/s41539-024-00249-3 (PMC11139856; doi:10.1038/s41539-024-00249-3)
Supplement: Supplementary file 1 — Supplementary Information [file 41539_2024_249_MOESM1_ESM.docx]

**SUPPLEMENTARY INFORMATION**

**A brief tablet-based intervention benefits linguistic and communicative abilities in toddlers and preschoolers.**

Marcela Peña, Constanza Vásquez-Venegas, Patricia Cortés, Enrica Pittaluga, Mitzy Herrera, Esteban J. Pino, Raul G. Escobar, Ghislaine Dehaene-Lambertz, Pamela Guevara^.^

This PDF file includes:

Supplementary Figures 1 to 5

Supplementary Tables 1 to 13

**Supplementary Figure 1 | Schedule of the experimental protocol.** A pre-post equivalent group design was employed because of its ability to detect subtle differences between groups. Before implementation of the intervention, each child’s linguistic and communicative abilities were assessed using standardized assessment batteries (see Methods). Based on their scores in these assessments, their age, and their sex, the children were ranked and randomly assigned to either a Study or Control group. Children in the Study group then received the training intervention, while those in the Control group did not. After the entirety of the Study group’s training concluded, the same assessment batteries were conducted again for the participants in both groups, and the gain in each child’s ability was calculated. Finally, the intervention was applied to the Control group.

**
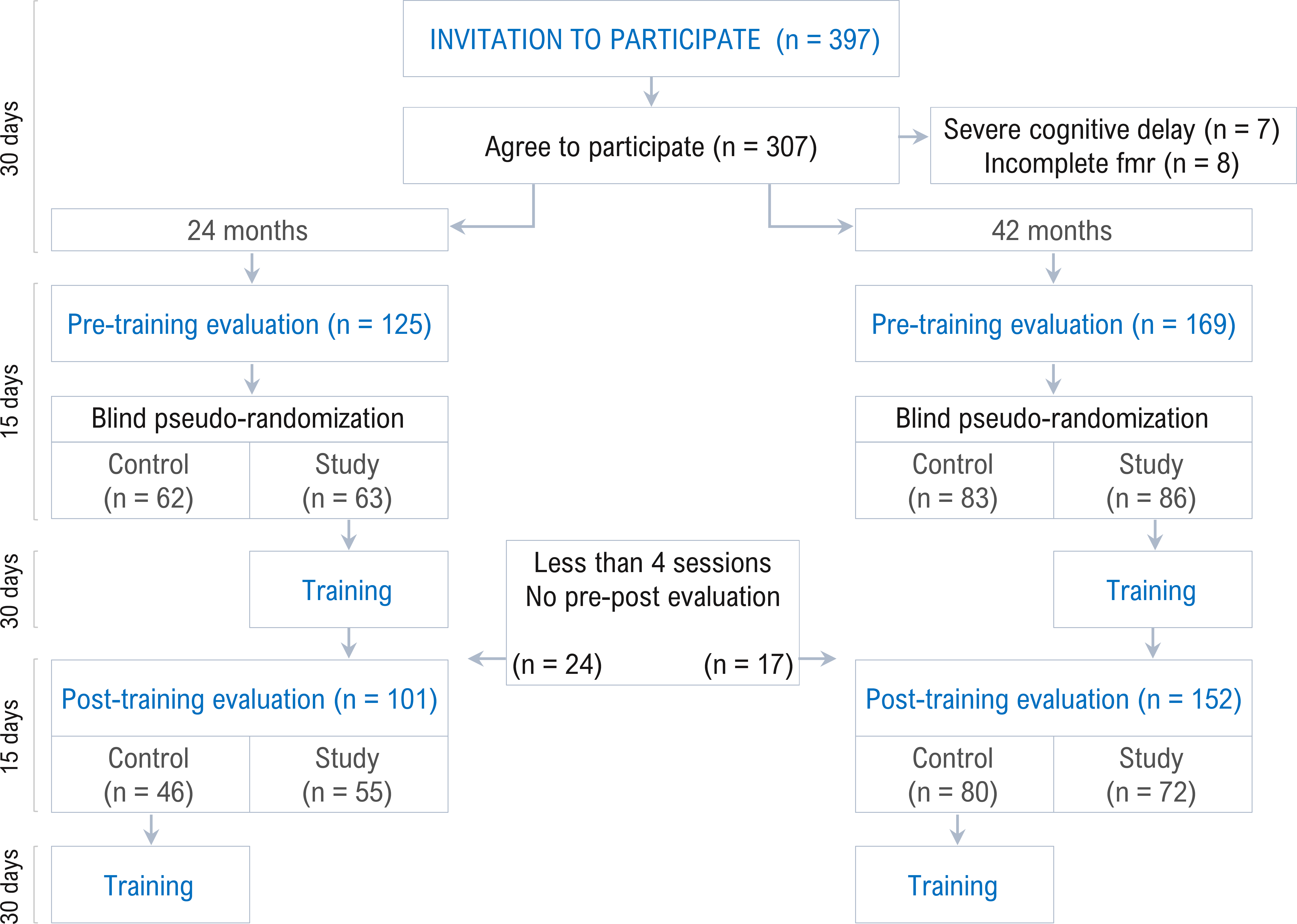
**

**Supplementary Figure 2 | The toddlers engaged with the game.** The toddlers consistently provided tactile responses in the majority of the game trials. We plot the proportion of non-omitted trials per task, phase, and group. Non-omitted trials include those where a tactile response was registered, regardless of its correctness. Each circle represents the data of one toddler. The asterisks indicate significant differences compared to the chance level, which corresponded to 0.5. ‘***’: *P* < 0.001.


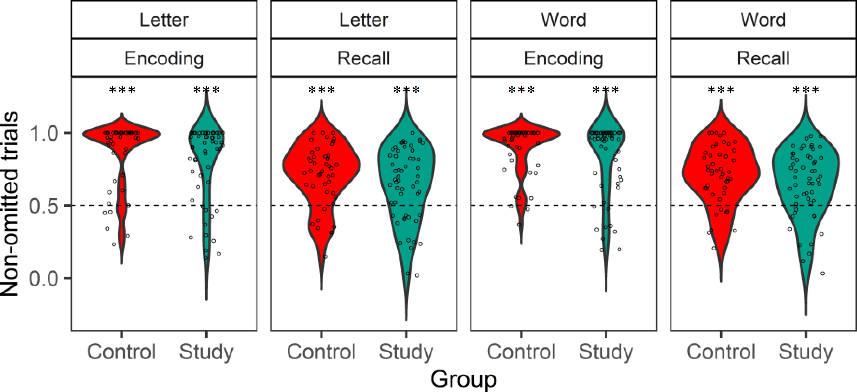


**Supplementary Figure 3 | All preschoolers engaged with the game.** The distribution of the proportion of the non-omitted trials per group (Control & Study), task (Letter-sound & Word-object association), and phase (Encoding, Recall & Vocalization) is graphed. Each circle corresponds to data of a preschooler computed over all sessions. The asterisks indicate significant differences against the chance level, which correspond to 0.5. ***: *P <* 0.001.


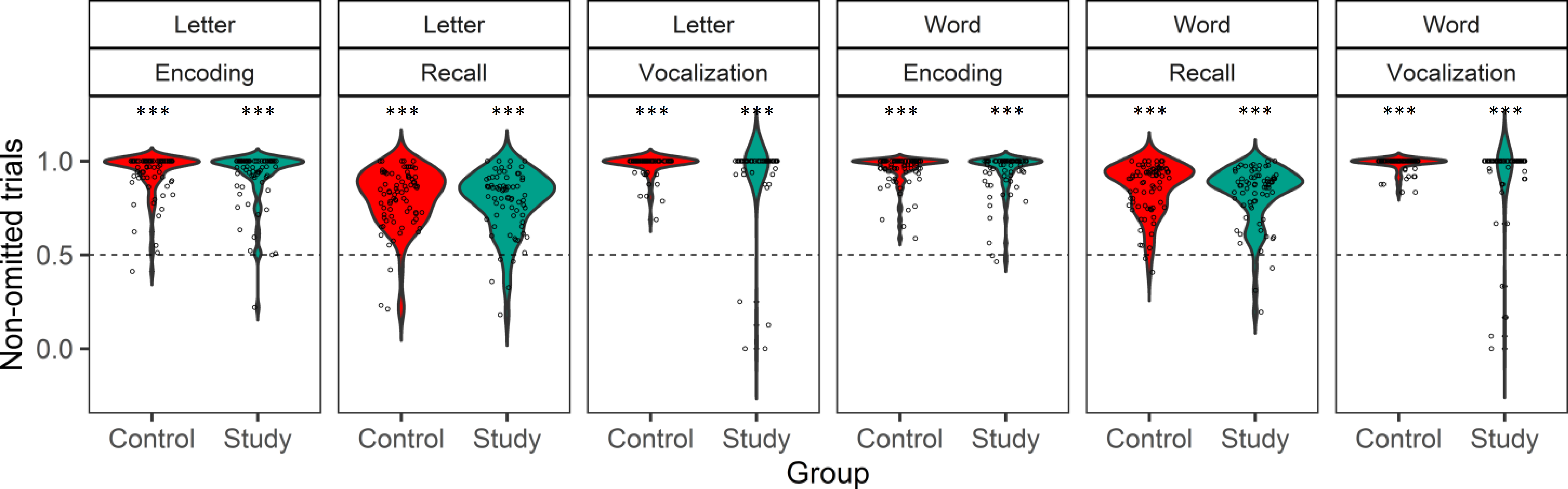


**Supplementary Figure 4** | **Bi-variate correlation between training performance and gain in linguistic skills**. Only the Study group’s accuracy in the Encoding phase of the Letter-sound trials was found to be positively correlated with the standardized gain in the TECAL total score**.**

**
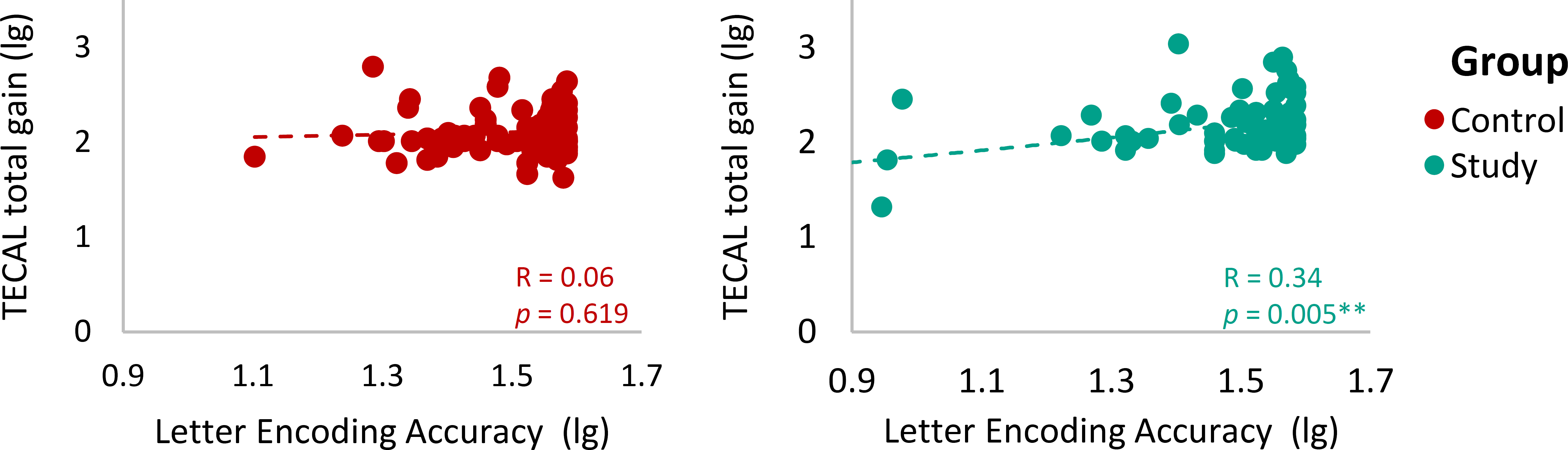
**

**Supplementary Figure 5 | Decision algorithm of the main app.** Decision algorithm of the main app. Each game session comprised 6 trials, which were structured in 3 phases: the encoding phase (yellow boxes), the recognition phase (orange boxes) presented to all children, and the vocalization phase (purple boxes) presented only to preschoolers. In the Encoding phase, a randomly chosen pair of words or letter-sounds were successively presented to the user in random locations (left or right side of the screen), and a video of the guiding educator called the child to touch the corresponding image. The user interaction with the app triggered three types of responses: correct, incorrect, and omitted, represented with green, red and blue arrows, respectively. In the Recognition phase, the app first randomly selected one of the two words or letter-sounds, and then simultaneously presented the two images presented in the encoding phase and a video of the guiding educator calling the child to touch the image that matched the selected word or letter-sound. We recorded the accuracy of each response. Finally, in the vocalization phase, the app requested the child to utter aloud the just evaluated word or letter-sound, and the supervising educator classified any word-like vocalization as correct and vocalizations such as screams, coughs, laughs, and similar vocalizations as incorrect. In each step of the game, the correct responses triggered positive feedback such as celebration videos and the game continued. The omitted responses triggered a second chance to answer. If there were two consecutive omissions or incorrect responses, the game skips to the next phase and delivered an encouraging message such as "Let´s try again". The timeout for tactile responses was 2 seconds in the encoding and recognition phases, and 7 seconds in the vocalization phase.


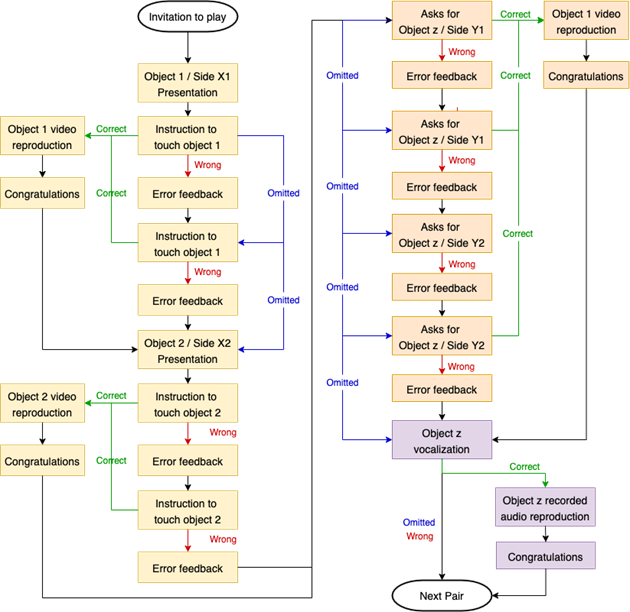


**Supplementary Table 1 | Linguistic and communicative gains in toddlers**. The Study group showed gains in ASQ-3’s Communication, Gross Motor, and Fine Motor domains, while the Control group only gained in ASQ-3’s Personal Social domain. In CDI, both groups gained in scores for the total and each subset of words during the evaluation period. The Wilcoxon signed rank test was applied to analyze the ASQ-3 data because they distributed non-normally, and two independent samples *t*-tests were applied to the CDI data because they distributed normally.

| **ASQ-3** | **Domain** | **Group** | **n** | **Median gain (lg)** | **Interquartile rank** | **W** | ***P*** | **Eff. size (r)** |
| --- | --- | --- | --- | --- | --- | --- | --- | --- |
|  | Communication | Control | 39 | 2.23 | 0.18 | 495 | 0.143 | 0.236 |
|  |  | Study | 53 | 2.32 | 0.27 | 1252 | < 0.001*** | 0.66 |
|  | Gross Motor | Control | 43 | 2.14 | 1.5 | 452 | 0.802 | 0.04 |
|  |  | Study | 53 | 2.14 | 1.6 | 986 | 0.013* | 0.21 |
|  | Fine Motor | Control | 43 | 2.29 | 0.27 | 568 | 0.252 | 0.17 |
|  |  | Study | 53 | 2.32 | 0.26 | 981 | 0.018* | 0.32 |
|  | Problem Solving | Control | 42 | 2.26 | 0.35 | 524 | 0.366 | 0.14 |
|  |  | Study | 51 | 2.14 | 0.32 | 756 | 0.384 | 0.123 |
|  | Personal Social | Control | 42 | 2.29 | 0.26 | 665 | 0.007** | 0.41 |
|  |  | Study | 51 | 2.29 | 0.27 | 839 | 0.098 | 0.23 |
| Wilcoxon signed rank test. ‘***’ = 0.001 ‘**’ = 0.01 ‘*’ = 0.05. Null gain corresponded to 2.14 in log format | | | | | | | | |
|  | | | | | | | | |
| **CDI** | **Type of words** | **Group** | **n** | **Mean gain (lg)** | **CI 95%** | ***t*** | ***P*** | **Cohen’ d** |
|  | Adverb Verb | Control | 45 | 1.38 | 0.11 | 6.64 | < 0.001*** | 0.99 |
|  |  | Study | 52 | 1.54 | 0.10 | 10.03 | < 0.001*** | 1.39 |
|  | Function | Control | 45 | 1.41 | 0.12 | 6.93 | < 0.001*** | 1.03 |
|  |  | Study | 52 | 1.35 | 0.12 | 5.53 | < 0.001*** | 0.77 |
|  | Noun Adjective | Control | 45 | 1.39 | 0.12 | 6.39 | < 0.001*** | 0.95 |
|  |  | Study | 52 | 1.39 | 0.11 | 6.81 | < 0.001*** | 0.94 |
|  | Sentence Complexity | Control | 45 | 1.17 | 0.12 | 2.55 | 0.014* | 0.38 |
|  |  | Study | 52 | 1.23 | 0.11 | 3.83 | < 0.001*** | 0.53 |
|  | Total | Control | 45 | 1.41 | 0.11 | 7.42 | < 0.001*** | 1.11 |
|  |  | Study | 52 | 1.44 | 0.10 | 8.59 | < 0.001*** | 1.19 |
| One sample *t*-test. ‘***’ 0.001 ‘**’ 0.01 ‘*’ 0.05. Null gain corresponded to 1.01 in log format. | | | | | | | | |

**Supplementary Table 2 | Robustbase multiple regression and ANCOVA in Experiment 1.**

*a) Robustbase multiple regression*. The dependent variables, standardized gain in the ASQ-3 Communication domain and in the CDI Adverbs-Verbs subscale were submitted to two robustbase multiple regression analyses, one with Group, Toddler’s age and Toddler’s sex, and another with Group, Mother’s age and Mother’s education as predictors. The models did not show any significant effect of Toddler’s or Mother’s data. In contrast, the factor Group did explain the greater gains found in the Study group.

| **ASQ Communication: Group, Toddler’s age and Toddler’s sex** | | | | | |
| --- | --- | --- | --- | --- | --- |
| **lmrob(formula = Communication_Gain ~ Group + Toddler_Age + Toddler_Sex, data = 24months)** | | | | | |
| Coefficients: |  |  |  |  |  |
|  | Estimate | Std. Error | t value | Pr(>\|t\|) |  |
| (Intercept) | 2.54 | 0.15 | 16.41 | <2e-16 *** |  |
| Group Study | 0.08 | 0.04 | 2.17 | 0.032 * |  |
| Toddler’s age | -0.01 | 0.01 | -1.92 | 0.056 |  |
| Toddler’s sex male | 0.01 | 0.04 | 0.34 | 0.733 |  |
|  |  |  |  |  |  |
| Robustness weights: |  |  |  |  |  |
| Min. | 1st Qu. | Median | Mean | 3rd Qu. | Max. |
| 0.13 | 0.86 | 0.95 | 0.88 | 0.98 | 1.00 |
| **ASQ Communication: Group, Mother’s age and Mother’s education** | | | | | |
| **lmrob(formula = Communication_Gain ~ Group + Mother_Age + Mother_Education, data = 24months)** | | | | | |
| Coefficients: |  |  |  |  |  |
|  | Estimate | Std. Error | t value | Pr(>\|t\|) |  |
| (Intercept) | 2.35 | 0.11 | 21.35 | <2e-16*** |  |
| Group study | 0.08 | 0.04 | 2.01 | 0.047* |  |
| Mother’s age | 0.00 | 0.00 | -1.20 | 0.235 |  |
| Mother’s education | -0.01 | 0.01 | -0.69 | 0.491 |  |
|  |  |  |  |  |  |
| Robustness weights: |  |  |  |  |  |
| Min. | 1st Qu. | Median | Mean | 3rd Qu. | Max. |
| 0.14 | 0.87 | 0.94 | 0.89 | 0.98 | 1.00 |
| **CDI AdverbVerb: Group, Toddler’s age and Toddler’s sex** | | | | | |
| **lmrob(formula = AdverbVerb_Gain ~ Group + Toddler_Age + Toddler_Sex, data = 24months)** | | | | | |
| Coefficients: |  |  |  |  |  |
|  | Estimate | Std. Error | t value | Pr(>\|t\|) |  |
| (Intercept) | 1.80 | 0.32 | 5.55 | 0.000*** |  |
| Group study | 0.14 | 0.08 | 2.17 | 0.043* | . |
| Toddler’s age | -0.02 | 0.01 | -1.51 | 0.134 |  |
| Toddler’s sex male | 0.07 | 0.08 | 0.89 | 0.376 |  |
|  |  |  |  |  |  |
| Robustness weights: |  |  |  |  |  |
| Min. | 1st Qu. | Median | Mean | 3rd Qu. | Max. |
| 0.16 | 0.83 | 0.95 | 0.88 | 0.98 | 1.00 |
| **CDI AdverbVerb: Group, Mother’s age and Mother’s education** | | | | | |
| **lmrob(formula = AdverbVerb_Gain ~ Group + Mother_Age + Mother_Education, data = 24months)** | | | | | |
| Coefficients: |  |  |  |  |  |
|  | Estimate | Std. Error | t value | Pr(>\|t\|) |  |
| (Intercept) | 1.44 | 0.33 | 4.31 | 0.000*** |  |
| Group study | 0.15 | 0.08 | 2.18 | 0.045* | . |
| Mother’s age | -0.01 | 0.01 | -0.54 | 0.594 |  |
| Mother’s education | 0.02 | 0.02 | 0.77 | 0.441 |  |
|  |  |  |  |  |  |
| Robustness weights: |  |  |  |  |  |
| Min. | 1st Qu. | Median | Mean | 3rd Qu. | Max. |
| 0.18 | 0.85 | 0.95 | 0.89 | 0.98 | 1.00 |
| Significance codes: 0 ‘***’ 0.001 ‘**’ 0.01 ‘*’ 0.05. Uncorrected *P*-values for planned comparisons. | | | | | |

*b) ANCOVA.* With ANCOVA, the possibility that the Study and Control groups already differed at the pre-training evaluation in the ASQ-3 Communication and the CDI Adverbs and Verbs raw scores was rejected. Non-parametric ANCOVA (test of equality) was applied to both data for comparison purposes. The results confirmed that the higher gains observed in the Study group appeared after the training.

|  |  |  | **Pre-training score** | | **Standardized gain** | |  |
| --- | --- | --- | --- | --- | --- | --- | --- |
| **Test of equality** | **Group** | **n** | **Median (lg)** | **Interquartile rank** | **Median (lg)** | **Interquartile rank** | ***P*** |
| ASQ-3 Communication | Control | 39 | 5.52 | 1.13 | 2.23 | 0.18 | 0.179 |
|  | Study | 53 | 5.17 | 0.72 | 2.32 | 0.27 |  |
| CDI Adverbs and Verbs | Control | 45 | 7.73 | 1.06 | 1.38 | 0.11 | 0.949 |
|  | Study | 52 | 7.83 | 0.915 | 1.54 | 0.1 |  |

**Supplementary Table 3 | Nonparametric longitudinal data analyses of accuracy by phase**. Effect of Phase in (**a)** the Letter-sound task and (**b)** the Word-object task in Experiment 1. The model includes the factors Group (Control & Study), Session (1 to 6), and Phase (Encoding (Enc) & Recognition (Rec)). '***' = p < 0.001. ':' represents interactions. ­


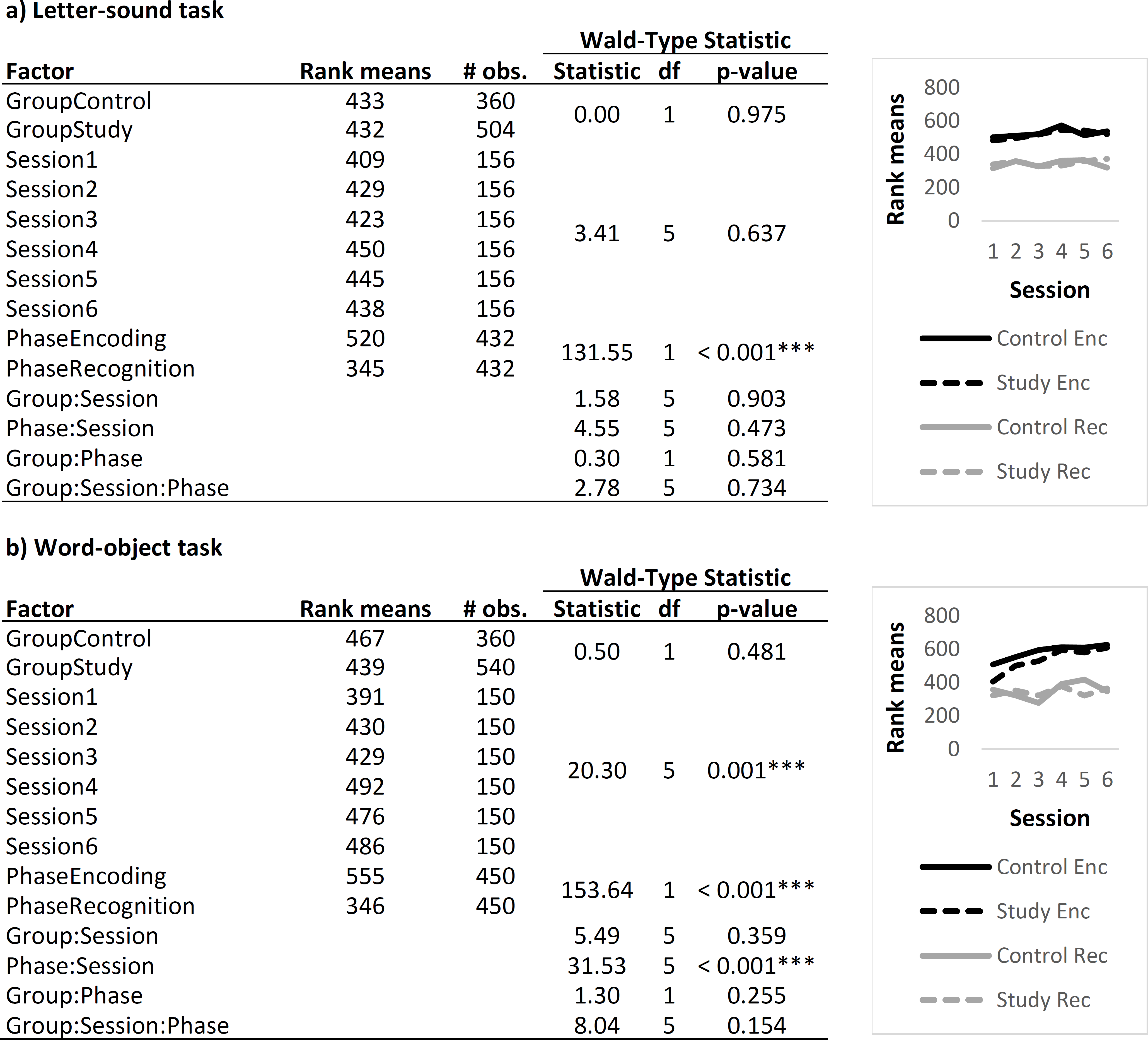


**Supplementary Table 4 | Nonparametric longitudinal data analysis of accuracy by task**. Effect of task in (**a)** the Encoding phase and **(b)** the Recording phase in Experiment 1. The model includes the factors Group (Control & Study), Session (1 to 6), and Task (Letter-sound & Word-object). '*' = p < 0.05. ':' represents interactions.


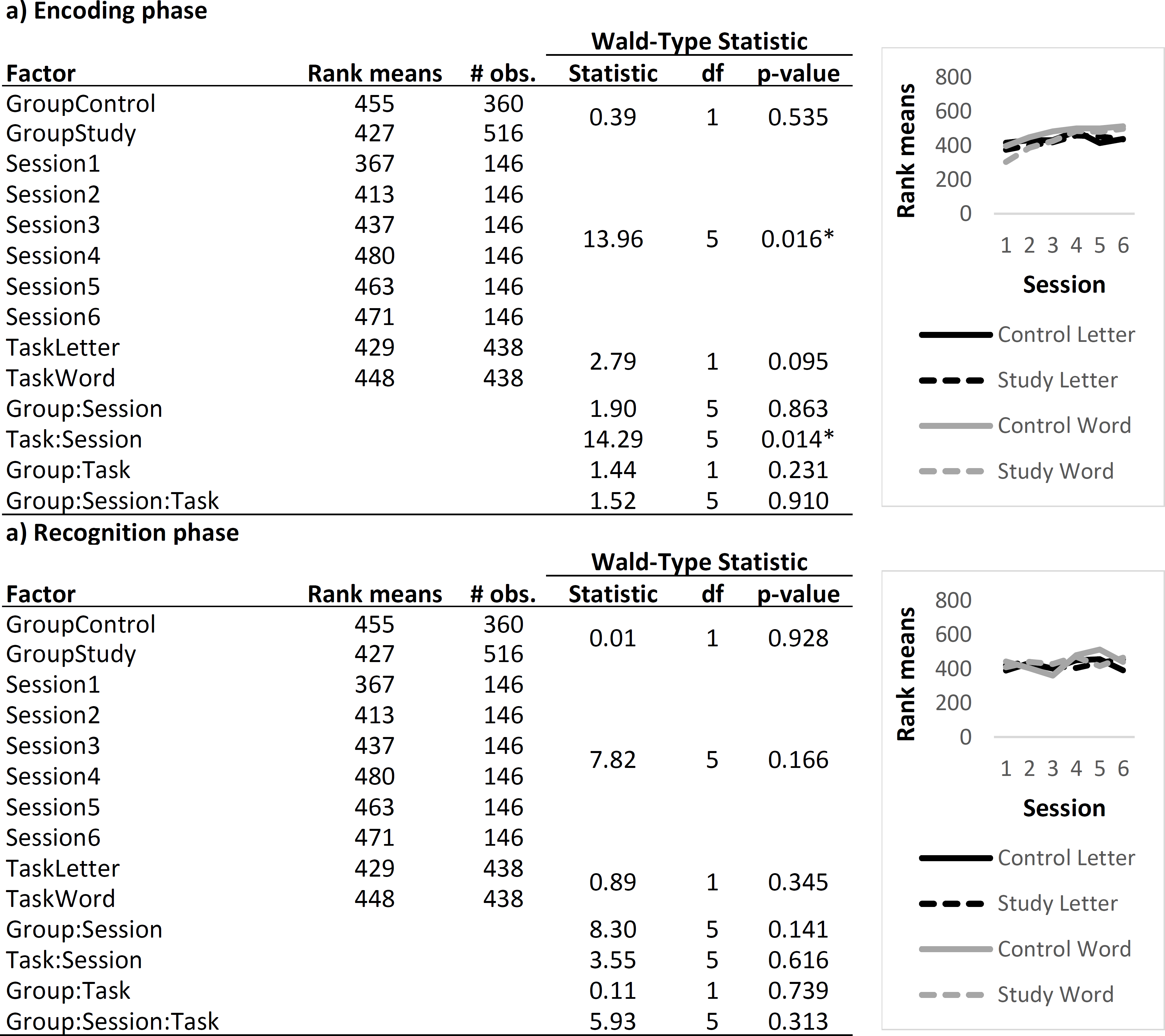


**Supplementary Table 5 | Gains against zero in preschoolers.**

**a) *ASQ-3****.*  Both groups showed a gain significantly higher than zero in the Gross Motor domain.

| **Domain** | **Group** | **n** | **Median gain (lg)** | **Interquartil rank** | **W** | ***P*** | **Effect size (r)** |
| --- | --- | --- | --- | --- | --- | --- | --- |
| Communication | Control | 70 | 2.14 | 0.19 | 1416 | 0.309 | 0.12 |
|  | Study | 61 | 2.14 | 0.28 | 1018 | 0.603 | 0.07 |
| Fine Motor | Control | 70 | 2.29 | 0.27 | 1567 | 0.057 | 0.29 |
|  | Study | 61 | 2.14 | 0.44 | 995 | 0.557 | 0.07 |
| Gross Motor | Control | 70 | 2.14 | 0.29 | 1592 | 0.039* | 0.25 |
|  | Study | 61 | 2.14 | 0.15 | 1224 | 0.044* | 0.26 |
| Personal Social | Control | 70 | 2.14 | 0.29 | 1491 | 0.221 | 0.15 |
|  | Study | 61 | 2.29 | 0.36 | 1078 | 0.229 | 0.16 |
| Problem Solving | Control | 70 | 2.14 | 0.24 | 1433 | 0.177 | 0.16 |
|  | Study | 61 | 2.14 | 0.12 | 1047 | 0.221 | 0.16 |
| Wilcoxon signed rank test. Significance codes: 0 ‘***’ 0.001 ‘**’ 0.01 ‘*’ 0.05. | | | | | | | |

**b) *TECAL and TEPROSIF****.* Both groups showed a gain significantly higher than zero in raw data of the TECAL Vocabulary sub-scale and total scores; and TEPROSIF, total score, indicating that all preschoolers improved in comprehensive and expressive linguistic measures during the intervention.

|  | **Linguistic ability** | **Group** | **n** | **Median gain (lg)** | **Interquartil rank** | **W** | ***P*** | **Effect size (r)** |
| --- | --- | --- | --- | --- | --- | --- | --- | --- |
| **TECAL** | Morphology | Control | 76 | 2.04 | 0.31 | 2041 | 0.003** | 0.34 |
|  |  | Study | 68 | 2.14 | 0.35 | 1841 | < 0.001*** | 0.49 |
|  | Syntax | Control | 76 | 2.01 | 0.32 | 1937 | 0.014* | 0.28 |
|  |  | Study | 68 | 2.01 | 0.31 | 1662 | 0.003** | 0.37 |
|  | Vocabulary | Control | 76 | 2.01 | 0.20 | 2034 | 0.003** | 0.34 |
|  |  | Study | 68 | 2.15 | 0.32 | 1993 | < 0.001*** | 0.61 |
|  | Total | Control | 76 | 2.07 | 0.23 | 2113 | < 0.001*** | 0.39 |
|  |  | Study | 68 | 2.13 | 0.30 | 2029 | < 0.001*** | 0.64 |
| **TEPROSIF** | Total | Control | 76 | 2.06 | 0.17 | 758 | <0.001*** | 0.42 |
|  |  | Study | 66 | 2.06 | 0.21 | 664 | < 0.005** | 0.35 |
| Wilcoxon signed rank test. Significance codes: 0 ‘***’ 0.001 ‘**’ 0.01 ‘*’ 0.05. | | | | | | | | |

**Supplementary Table 6 | Robustbase multiple regression and ANCOVA in Experiment 2.**

***a) Robustbase multiple regression analysis*.**  The standardized gain in the TECAL Vocabulary subscale and TECAL total score were submitted to two robustbase multiple regression analyses, one with Group, Preschooler’s age and Preschooler’s sex and another with Group, Mother’s age and Mother’s education as predictors. As in Experiment 1, the models did not show significant effects of Preschooler’s or Mother’s factors, while the factor Group explained the greater gains found in the Study group.

| **TECAL gain in vocabulary by Group, Preschool’s age and Preschool’s sex** | | | | | |
| --- | --- | --- | --- | --- | --- |
| **lmrob(formula = Vocabulary_Gain ~ Group + Preschool_Age + Preschool_Sex, data = 42months)** | | | | | |
| Coefficients: |  |  |  |  |  |
|  | **Estimate** | **Std. Error** | **t value** | **Pr(>\|t\|)** |  |
| (Intercept) | 2.13 | 0.24 | 8.94 | 0.000*** |  |
| Group study | 0.08 | 0.04 | 2.10 | 0.037* |  |
| Preschool’s age | 0.00 | 0.01 | -0.28 | 0.777 |  |
| Preschool’s sex male | 0.00 | 0.04 | 0.06 | 0.952 |  |
|  |  |  |  |  |  |
| Robustness weights: |  |  |  |  |  |
| Min. | 1st Qu. | Median | Mean | 3rd Qu. | Max. |
| 0.00 | 0.87 | 0.95 | 0.87 | 0.98 | 1.00 |
| **TECAL Vocabulary: Group, Mother’s age and Mother’s education** | | | | | |
| **lmrob(formula = Vocabulary_Gain ~ Group + Mother_Age + Mother_Education, data = 42months)** | | | | | |
| Coefficients: |  |  |  |  |  |
|  | **Estimate** | **Std. Error** | **t value** | **Pr(>\|t\|)** |  |
| (Intercept) | 1.76 | 0.17 | 10.13 | 0.000 |  |
| Group study | 0.15 | 0.07 | 2.01 | 0.049* |  |
| Mother’s age | 0.01 | 0.01 | 1.81 | 0.075 |  |
| Mother’s education | 0.01 | 0.01 | 0.66 | 0.514 |  |
|  |  |  |  |  |  |
| Robustness weights: |  |  |  |  |  |
| Min. | 1st Qu. | Median | Mean | 3rd Qu. | Max. |
| 0.20 | 0.87 | 0.96 | 0.90 | 0.98 | 1.00 |
| **TECAL total score: Group, Preschool’s age and Preschool’s sex** | | | | | |
| **lmrob(formula = Total_Gain ~ Group + Preschool_Age + Preschool_Sex, data = 42months)** | | | | | |
| Coefficients: |  |  |  |  |  |
|  | **Estimate** | **Std. Error** | **t value** | **Pr(>\|t\|)** |  |
| (Intercept) | 2.07 | 0.27 | 7.77 | 0.000*** |  |
| Group study | 0.08 | 0.04 | 2.04 | 0.043*** |  |
| Preschool’s age | 0.00 | 0.01 | 0.09 | 0.930 |  |
| Preschool’s sex male | -0.03 | 0.04 | -0.67 | 0.504 |  |
|  |  |  |  |  |  |
| Robustness weights: |  |  |  |  |  |
| Min. | 1st Qu. | Median | Mean | 3rd Qu. | Max. |
| 0.00 | 0.85 | 0.95 | 0.87 | 0.98 | 1.00 |
| **TECAL total score: Group, Mother’s age and Mother’s education** | | | | | |
| **lmrob(formula = Total_Gain ~ Group + Mother_Age + Mother_Education, data = 42months)** | | | | | |
| Coefficients: |  |  |  |  |  |
|  | **Estimate** | **Std. Error** | **t value** | **Pr(>\|t\|)** |  |
| (Intercept) | 1.72 | 0.19 | 9.14 | 0.000*** |  |
| Group study | 0.01 | 0.01 | 2.20 | 0.031* |  |
| Mother’s age | 0.10 | 0.07 | 1.40 | 0.166 |  |
| Mother’s education | 0.008 | 0.015 | 0.549 | 0.584 |  |
|  |  |  |  |  |  |
| Robustness weights: |  |  |  |  |  |
| Min. | 1st Qu. | Median | Mean | 3rd Qu. | Max. |
| 0.04 | 0.82 | 0.96 | 0.86 | 0.99 | 1.00 |
| Signif. codes: 0 ‘***’ 0.001 ‘**’ 0.01 ‘*’ 0.05. Uncorrected *P*-values reported for planned comparisons. | | | | | |

**b) *ANCOVA*.** With ANCOVA analysis, the possibility that the Study and Control groups already differed at the pre-training evaluation in the TECAL Vocabulary subscale score and total score was rejected. Non-parametric ANCOVA (test of equality) was applied because data were non-normally distributed. The results showed that Control and Study groups were similar before the intervention.

|  |  |  | **Pre-training** | | **Standardized gain** | |  |
| --- | --- | --- | --- | --- | --- | --- | --- |
| **Linguistic abilities** | **Group** | **N** | **Median gain (lg)** | **Interquartile rank** | **Median gain (lg)** | **Interquartile rank** | ***P*** |
| TECAL Vocabulary | Control | 76 | 4.76 | 0.263 | 2.01 | 0.20 | 0.885 |
|  | Study | 68 | 4.7 | 0.322 | 2.15 | 0.32 |  |
| TECAL Total | Control | 76 | 5.81 | 0.408 | 2.07 | 0.23 | 0.531 |
|  | Study | 68 | 5.73 | 0.339 | 2.13 | 0.3 |  |

**Supplementary Table 7 | Nonparametric longitudinal data analysis of accuracy in Experiment 2 by phase**. Effect of Phase in (**a**) the Letter-sound task and (**b**) the Word-object task. The model’s factors are Group (Control & Study), Session (1 to 6), and Phase (Encoding & Recognition). '***' = p < 0.001. ':' represents interactions.


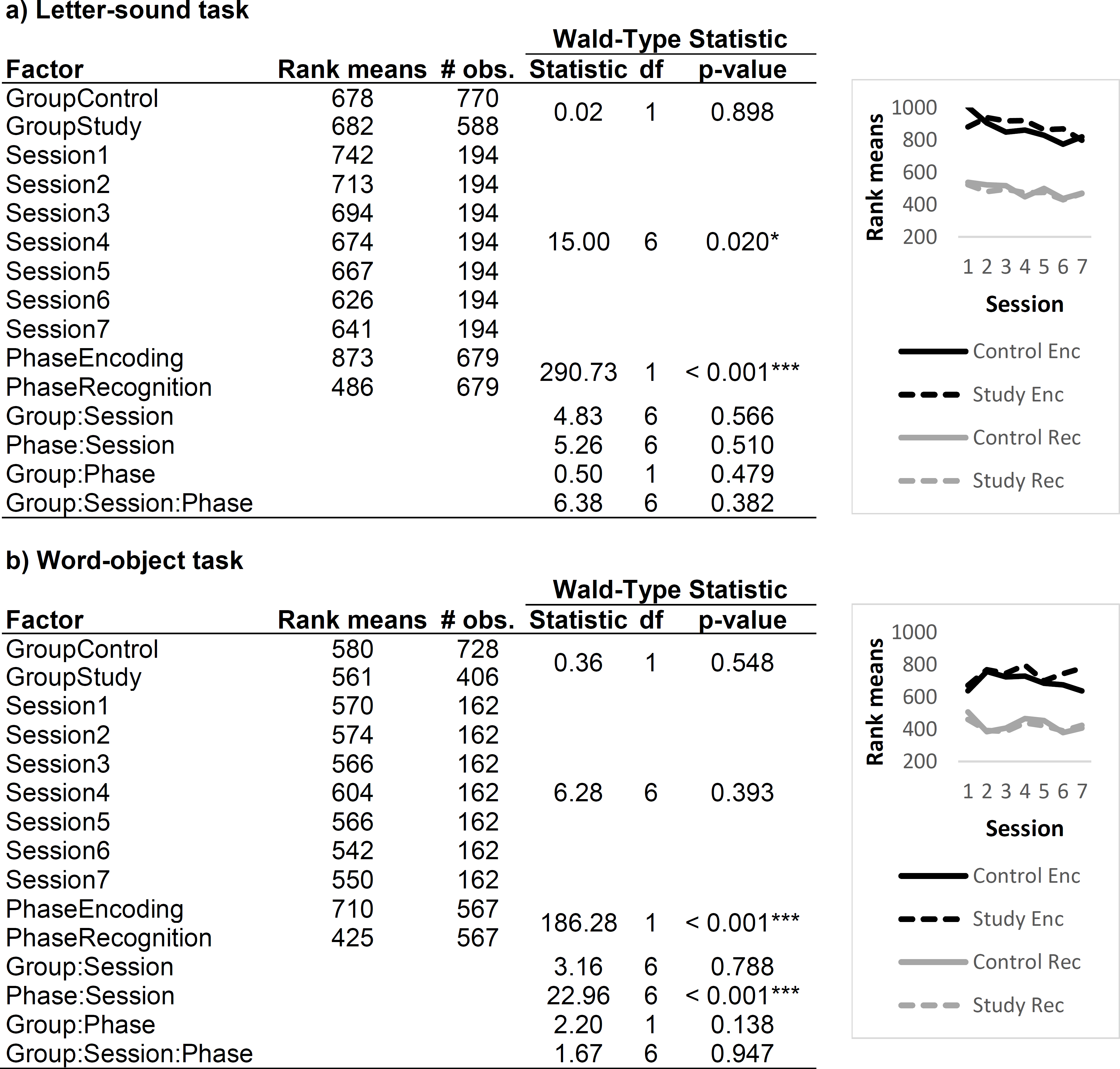


**Supplementary Table 8 | Nonparametric longitudinal data analysis of accuracy by task**. Effect of Task in **(a)** the Encoding, **(b)** Recognition, and **(c)** Vocalization phases in Experiment 2. The factors were Group (Control & Study), Session (1 to 7), and Task (Word-object & Letter-sound). '***' = p < 0.001. ':' represents interactions.


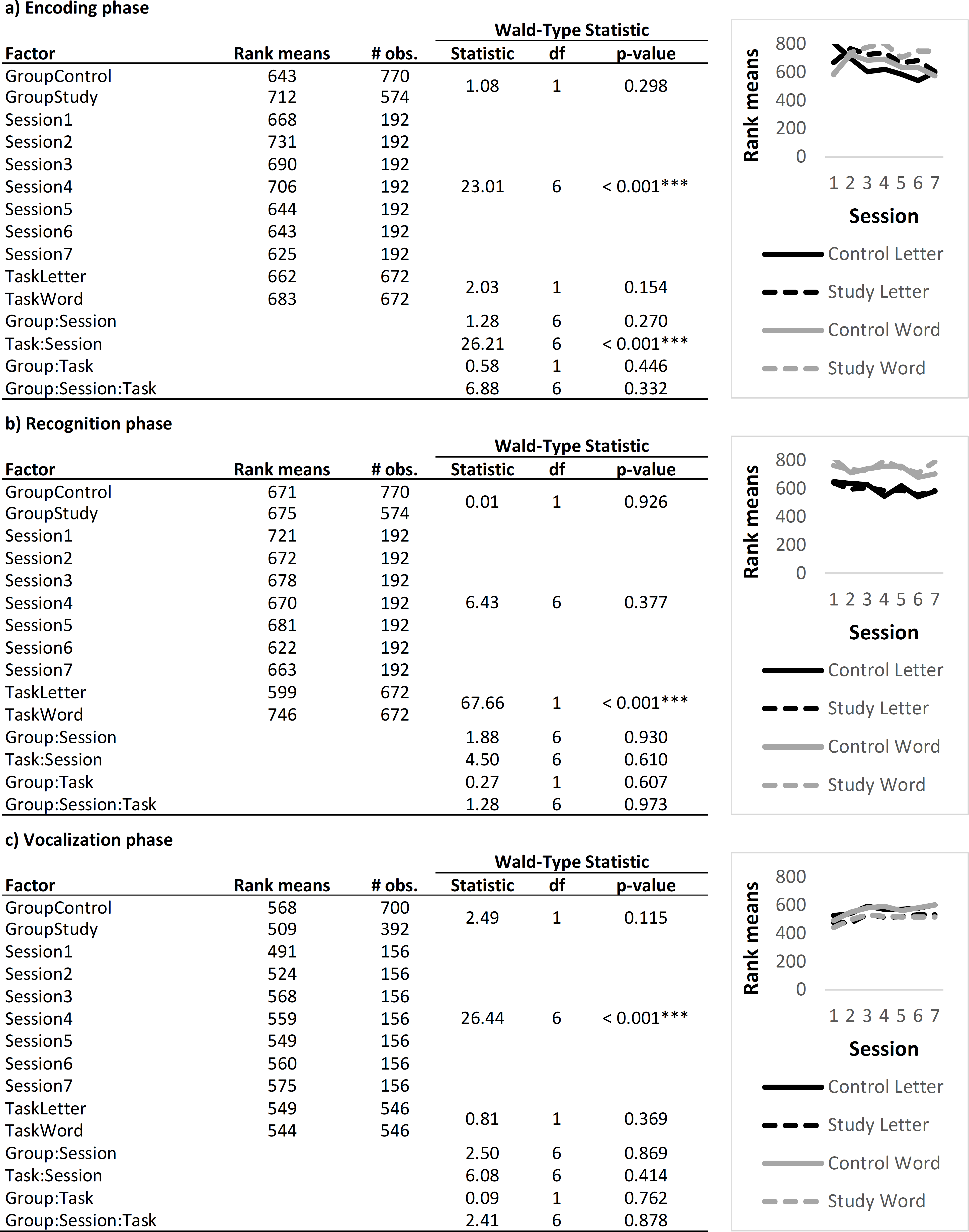


**Supplementary Table 9 | Nonparametric longitudinal data analysis of accuracy in both experiments for letter-sound task in each phase**. The model’s factors were Experiment (Exp1 & Exp2), Group (Control & Study), and Session (1 to 7). '***' = p < 0.001. ':' represents interactions.


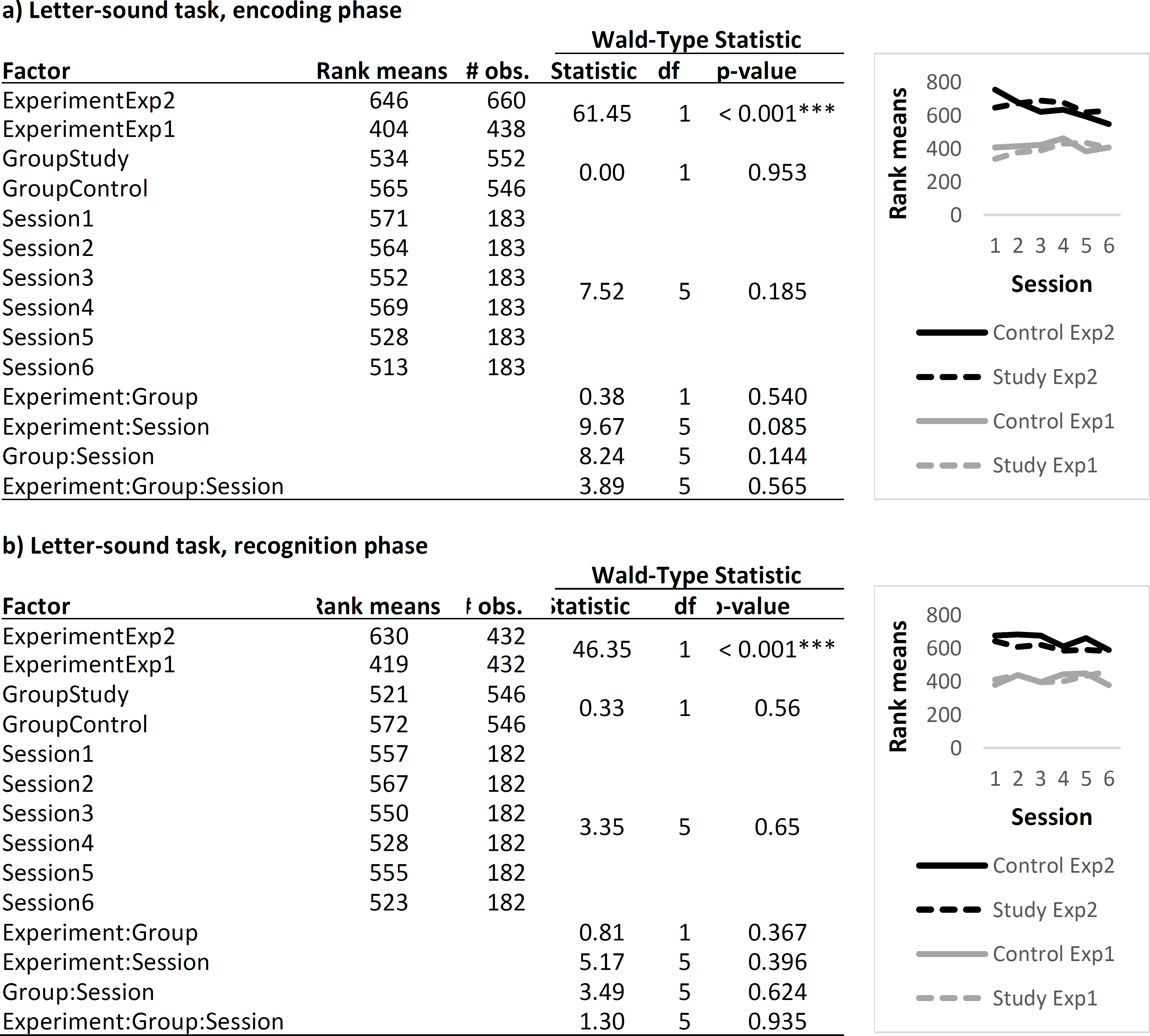


**Supplementary Table 10 | Nonparametric longitudinal data analysis of accuracy in both experiments for word-object task in each phase**. The model’s factors were Experiment (Exp1 & Exp2), Group (Control & Study), and Session (1 to 6). '***' = p < 0.001. ':' represents interactions.


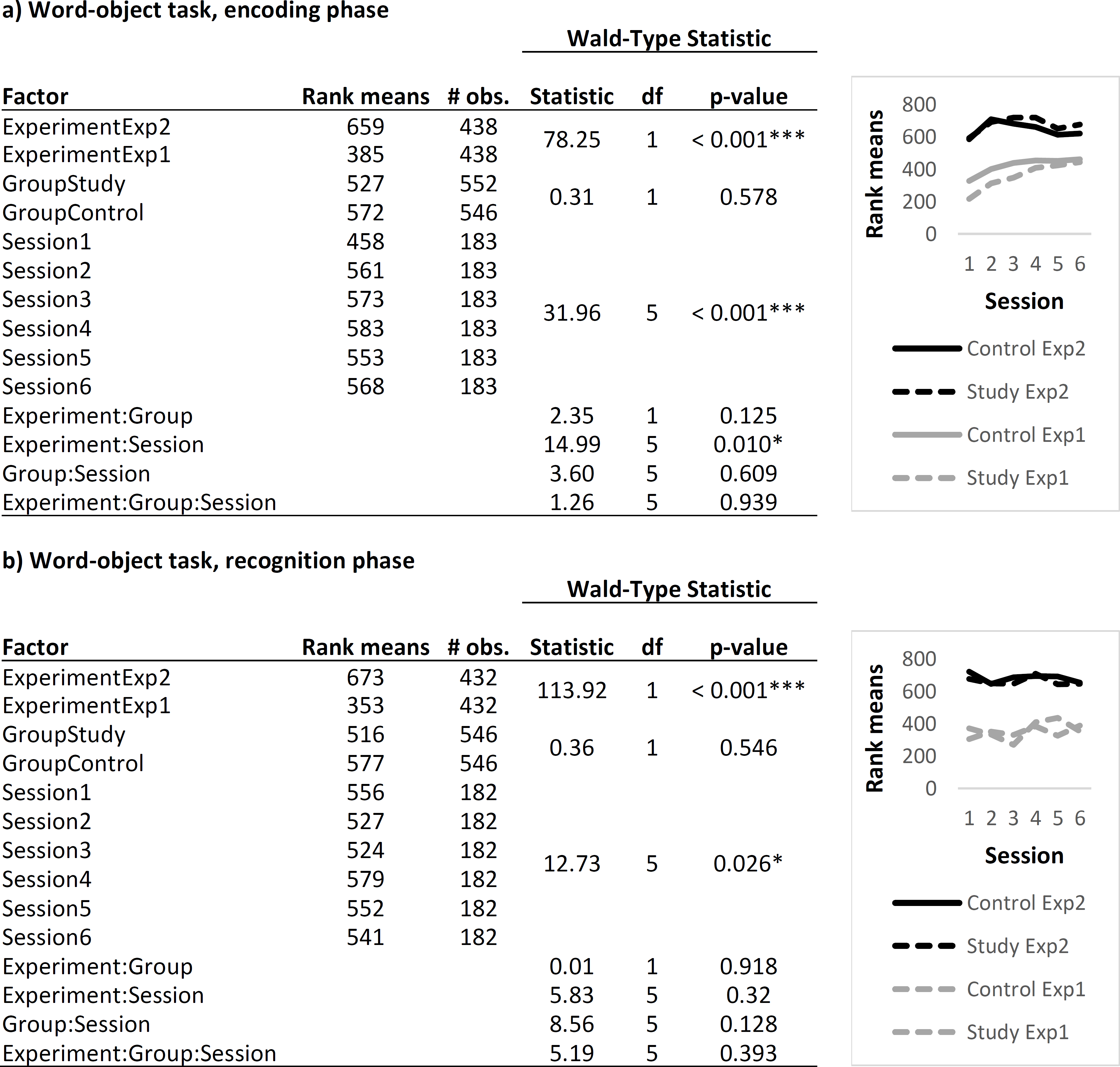


**Supplementary Table 11 | Toddlers’ pre-training data.** After attrition, the remaining children of the Control and Study groups did not differ in their bio-demographic data, or their linguistic and communicative abilities assessed at the pre-training evaluation. The number of participants (n) varies across the analyzed data because some parents did not provide full information in questionnaires or because some toddler’s assessments were inconclusive.

| **Variable** | **Group** | **N** | **Median** | **Interquartile rank** | **Z** | ***P*** | **Effect size (r)** |
| --- | --- | --- | --- | --- | --- | --- | --- |
| Toddler’s age | Control | 46 | 23 | 3.28 | 0.20 | 0.843 | 0.02 |
|  | Study | 55 | 23 | 4.6 |  |  |  |
| Toddler’s sex^1^ | Control | 46 | 2 | 1 | 0.23 | 0.842 | 0.02 |
|  | Study | 55 | 2 | 1 |  |  |  |
| Mother’s age | Control | 42 | 31 | 8 | 1.34 | 0.182 | 0.13 |
|  | Study | 55 | 30 | 7 |  |  |  |
| Mother’s education code^2^ | Control | 42 | 4 | 0.75 | -1.74 | 0.082 | -0.17 |
|  | Study | 55 | 4 | 2 |  |  |  |
| Father Age^3^ | Control | 25 | 35 | 12 | 0.18 | 0.857 | 0.02 |
|  | Study | 43 | 31 | 12.5 |  |  |  |
| Father Education code^2,3^ | Control | 25 | 4 | 3 | -1.11 | 0.274 | -0.11 |
|  | Study | 43 | 4 | 2 |  |  |  |
| ASQ Communication | Control | 46 | 45 | 23.8 | 0.52 | 0.605 | 0.05 |
|  | Study | 55 | 40 | 20 |  |  |  |
| ASQ Gross Motor | Control | 46 | 55 | 13.8 | 0.15 | 0.882 | 0.01 |
|  | Study | 54 | 55 | 15 |  |  |  |
| ASQ Fine Motor | Control | 46 | 50 | 10 | 0.36 | 0.723 | 0.03 |
|  | Study | 54 | 45 | 13.7 |  |  |  |
| ASQ Problem Solving | Control | 46 | 45 | 10 | 0.09 | 0.921 | 0 |
|  | Study | 54 | 45 | 13.8 |  |  |  |
| ASQ Personal Social | Control | 46 | 47.5 | 15 | 0.96 | 0.342 | 0.09 |
|  | Study | 54 | 45 | 18.8 |  |  |  |
| CDI Sentence Complexity | Control | 45 | 25 | 6 | 0.63 | 0.533 | 0.06 |
|  | Study | 53 | 25 | 6 |  |  |  |
| CDI Nouns Adjectives | Control | 45 | 149 | 114 | -0.43 | 0.666 | -0.04 |
|  | Study | 53 | 170 | 109 |  |  |  |
| CDI Adverbs Verbs | Control | 45 | 39 | 35 | 0.47 | 0.641 | 0.05 |
|  | Study | 53 | 37 | 31 |  |  |  |
| CDI Function | Control | 45 | 17 | 36 | -0.46 | 0.643 | -0.04 |
|  | Study | 53 | 24 | 30 |  |  |  |
| CDI Total | Control | 45 | 211 | 158 | -0.45 | 0.656 | -0.04 |
|  | Study | 53 | 229 | 137 |  |  |  |

^1^Toddler’s sex data were converted into numeric for statistical comparison. Female corresponded to 1 and male to 2; ^2^Education level data were converted into numeric for statistical comparison: 0 illiterate; 1 =1 to 4 years; 2 = 5 to 8 years; 3 =9 to 11 years; 4 = 12 years; 5 = professional 13 to 14 years; 6 = university 13 to 16 years; 7 university 17 or 19 years; 8 = postgraduate (more than 19 years); ^3^Many fathers did not provide age and educational level data, we did not further report their data.

**Supplementary Table 12 |** **Preschoolers’ pre-training data.** The preschoolers of the Control and Study groups did not significantly differ in their bio-demographic data or linguistic data in the pre-training evaluation.

| **Variable** | **Group** | **N** | **Median** | **Interquartile rank** | **Z** | ***P*** | **Effect size (r)** |
| --- | --- | --- | --- | --- | --- | --- | --- |
| Preschooler’s age | Control | 80 | 39.2 | 4.95 | 0.06 | 0.954 | 0.00 |
|  | Study | 72 | 39.7 | 4.9 |  |  |  |
| Preschooler’s sex code^1^ | Control | 80 | 2 | 1 | -0.10 | 1.00 | 0.00 |
|  | Study | 72 | 2 | 1 |  |  |  |
| Mother’s age | Control | 40 | 35 | 9.25 | 1.07 | 0.289 | 0.08 |
|  | Study | 33 | 30 | 5 |  |  |  |
| Mother’s education code^2^ | Control | 37 | 4 | 1 | -0.54 | 0.594 | -0.04 |
|  | Study | 31 | 4 | 1 |  |  |  |
| Father Age^3^ | Control | 32 | 35 | 12.2 | 0.39 | 0.704 | 0.03 |
|  | Study | 22 | 33 | 8.5 |  |  |  |
| Father Educaction code^2,3^ | Control | 30 | 4 | 1 | -0.97 | 0.338 | -0.08 |
|  | Study | 20 | 4 | 1 |  |  |  |
| ASQ Communication | Control | 79 | 50 | 10 | -0.78 | 0.440 | -0.06 |
|  | Study | 71 | 55 | 15 |  |  |  |
| ASQ Gross Motor | Control | 79 | 55 | 10 | -0.35 | 0.723 | -0.03 |
|  | Study | 72 | 55 | 10 |  |  |  |
| ASQ Fine Motor | Control | 79 | 45 | 17.2 | -0.65 | 0.519 | -0.05 |
|  | Study | 72 | 45 | 21.2 |  |  |  |
| ASQ Problem Solving | Control | 79 | 50 | 15 | -1.19 | 0.238 | .0.09 |
|  | Study | 71 | 55 | 12.5 |  |  |  |
| ASQ Personal Social | Control | 80 | 50 | 10 | -0.08 | 0.939 | -0.01 |
|  | Study | 72 | 50 | 10 |  |  |  |
| TEPROSIF | Control | 76 | 46.5 | 24.2 | 0.85 | 0.397 | 0.07 |
|  | Study | 68 | 43.5 | 29.2 |  |  |  |
| TECAL vocabulary | Control | 77 | 26 | 5 | 1.02 | 0.306 | 0.08 |
|  | Study | 70 | 25 | 6 |  |  |  |
| TECAL morphology | Control | 77 | 24 | 9 | 0.99 | 0.325 | 0.08 |
|  | Study | 70 | 22.5 | 8 |  |  |  |
| TECAL syntax | Control | 77 | 6 | 2 | -0.02 | 0.983 | 0.00 |
|  | Study | 70 | 6 | 2.75 |  |  |  |
| TECAL total | Control | 77 | 55 | 16 | 1.02 | 0.311 | 0.08 |
|  | Study | 70 | 51.5 | 13 |  |  |  |

^1^Preschooler’s sex data were converted into numeric values for statistical comparison. Female corresponded to 1 and to 2; ^2^Education level data were converted into numeric values for statistical comparison: 0 = illiterate; 1 = 1 to 4 years; 2 = 5 to 8 years; 3 = 9 to 11 years; 4 = 12 years; 5 = professional 13 to 14 years; 6 = university 13 to 16 years; 7 = university 17 or 19 years; 8 = postgraduate (more than 19 years); ^3^As in Experiment 1, many fathers did not provide age and educational level, thus, we did not further report their data.

**Supplementary Table 13** | **List of stimuli**. The table shows the words in Spanish and their English translation, and the letters’ sounds evaluated in this study.

| **WORDS** | | | | | | | | | | | | **LETTERS** | |
| --- | --- | --- | --- | --- | --- | --- | --- | --- | --- | --- | --- | --- | --- |
| **#** | **Spanish** | **English** | **#** | **Spanish** | **English** | **#** | **Spanish** | **English** | **#** | **Spanish** | **English** | **#** | **Letter** |
| 1 | Alce | Moose | 16 | Tucan | Toucan | 31 | Bombo | Bass drum | 46 | Molino | Mill | 1 | A |
| 2 | Almeja | Clam | 17 | Vicuña | Vicuna | 37 | Canasta | Basket | 47 | Paleta | Paddle | 2 | E |
| 3 | Bisonte | Bison | 18 | Clavel | Carnation | 33 | Chala | Sandal | 48 | Pandero | Tambourine | 3 | I |
| 4 | Cabra | Goat | 19 | Hoja | Leaf | 34 | Clip | Clip | 49 | Patines | Skates | 4 | O |
| 5 | Cangrejo | Crab | 20 | Rosa | Rose | 35 | Cono | Cone | 50 | Pluma | Feather | 5 | U |
| 5 | Cóndor | Condor | 24 | Cebolla | Onion | 39 | Flauta | Flute | 54 | Trompo | Spinning top | 9 | L |
| 6 | Cebra | Zebra | 21 | Sauce | Willow | 36 | Dado | Die | 51 | Saxofón | Saxophone | 0 | G |
| 7 | Cisne | Swan | 22 | Trigo | Wheat | 37 | Dron | Drone | 52 | Silbato | Whistle | 7 | F |
| 8 | Colibrí | Hummingbird | 23 | Alfajor | Alfajor | 38 | Esfinge | Sphinx | 53 | Trompeta | Trumper | 8 | K |
| 10 | Foca | Seal | 25 | Kiwid | Kiwi | 40 | Gaita | Bagpipe | 55 | Tuba | Tuba | 10 | M |
| 11 | Garza | Heron | 26 | Mango | Mango | 41 | Gong | Gong | 56 | Tuerca | Nut | 9 | P |
| 12 | Morsa | Walrus | 27 | Nuez | Walnut | 42 | Grua | Crane | 57 | Velero | Sailboat | 12 | R |
| 13 | Narval | Narwhal | 28 | Piñon | Pinon | 43 | Guitarra | Guitar | 58 | Yoyo | Yoyo | 13 | S |
| 14 | Perdiz | Partridge | 75 | Seta | Mushroom | 44 | Lira | Lyre | 59 | Zueco | Clog | 14 | T |
| 15 | Pudu | Pudu | 30 | Arpa | Harp | 45 | Lupa | Magnifying glass |  |  |  |  |  |
